# Anaerobically Grown *Escherichia coli* Has an Enhanced Mutation Rate and Distinct Mutational Spectra

**DOI:** 10.1371/journal.pgen.1006570

**Published:** 2017-01-19

**Authors:** Sonal Shewaramani, Thomas J. Finn, Sinead C. Leahy, Rees Kassen, Paul B. Rainey, Christina D. Moon

**Affiliations:** 1 AgResearch Ltd, Grasslands Research Centre, Palmerston North, New Zealand; 2 New Zealand Institute for Advanced Study, Massey University, Auckland, New Zealand; 3 Department of Biology, University of Ottawa, Ottawa, Ontario, Canada; 4 Department of Microbial Population Biology, Max Planck Institute for Evolutionary Biology, Plön, Germany; 5 Ecole Supérieure de Physique et de Chimie Industrielles de la Ville de Paris (ESPCI ParisTech), CNRS UMR 8231, PSL Research University, Paris, France; University of Pittsburgh, UNITED STATES

## Abstract

Oxidative stress is a major cause of mutation but little is known about how growth in the absence of oxygen impacts the rate and spectrum of mutations. We employed long-term mutation accumulation experiments to directly measure the rates and spectra of spontaneous mutation events in *Escherichia coli* populations propagated under aerobic and anaerobic conditions. To detect mutations, whole genome sequencing was coupled with methods of analysis sufficient to identify a broad range of mutational classes, including structural variants (SVs) generated by movement of repetitive elements. The anaerobically grown populations displayed a mutation rate nearly twice that of the aerobic populations, showed distinct asymmetric mutational strand biases, and greater insertion element activity. Consistent with mutation rate and spectra observations, genes for transposition and recombination repair associated with SVs were up-regulated during anaerobic growth. Together, these results define differences in mutational spectra affecting the evolution of facultative anaerobes.

## Author Summary

When organisms are exposed to different environments, the rates and types of mutations that spontaneously arise in each environment can vary due to differing mutagenic pressures imposed by each environment, and these can potentially influence the evolution of the organism. Little is known about the types of mutations that arise when facultative anaerobes are grown in the absence of oxygen. To investigate the effect of oxygen availability on spontaneous mutation at the genome level, we directly measured the mutation rates of bacteria that were grown with and without oxygen under controlled laboratory conditions. To our knowledge, we have obtained the most comprehensive mutation rate estimate of anaerobically grown *Escherichia coli*, where mutation rates are higher in the absence of oxygen. We found that genomic structural variations, a class of mutations that are typically not investigated due to technical difficulties, were more prevalent under anaerobic growth conditions. We also identified distinct DNA strand biases for substitution types under aerobic and anaerobic growth conditions. Our findings provide new insights into the impact that oxygen availability may have on spontaneous mutations, and the mutational processes that underlie them.

## Introduction

Mutations are the ultimate source of genetic variation and play a central role in the tempo of evolution. Spontaneous mutation rates [1] vary considerably among different organisms [2–4] and grown in different environments [5]. Facultative anaerobes are metabolically versatile, being able to grow in both the presence and absence of oxygen, though the impact that each condition and contrasting cellular physiology has on spontaneous mutation rates and molecular spectra has received little attention [5].

Oxygen is the terminal electron acceptor during aerobic respiration with mutagenic reactive oxygen species (ROS) being normal cellular by-products. The impact of ROS on mutation rates and spectra has been documented [5, 6] with G →T transversions being a hallmark change [7]. However, in the absence of oxygen, energy can be generated by either anaerobic respiration or fermentation [8]. These metabolic modes result in different intracellular conditions that may impact spontaneous mutations. ROS concentrations in anaerobically grown cells are lower, thus less ROS-initiated mutations are expected to occur. In contrast, fermentation end-products typically include short-chain fatty acids, which may directly induce DNA damage, or be mutagenic through the acid stress response [9, 10]. Furthermore, compared to aerobically grown cells, anaerobically grown cells display slower growth rates as less ATP is generated per unit substrate.

*Escherichia coli* is one of the most extensively characterized facultative anaerobes, and can undergo anaerobic respiration in the presence of electron acceptors [8]. In the absence of such compounds, *E*. *coli* employs a “mixed acid” fermentation, where redox balance is achieved by the formation of a variety of end products [9]. *Escherichia coli* mutation rates and evolution have been extensively studied under aerobic conditions [6, 11–13]. Additionally, experimental systems that take advantage of its ability to rapidly attain large population sizes, ease of handling, and small genome size, have been established [13–15]. To measure spontaneous mutation rates, mutation accumulation (MA) experiments are commonly used [4, 6, 12], and genome re-sequencing allows for detailed genome-wide mutation detection [4, 16]. While base pair substitutions (BPSs) and insertions or deletions of one or few nucleotides (indels) are readily identifiable, structural variations (SVs) of the genome, such as chromosomal rearrangements, mobile genetic element (MGE) movement, MGE-mediated deletions, inversions and translocations of large genome regions are less readily detected. SVs generally arise from recombination between homologous or partially homologous DNA sequences, or the transposition of elements such as insertion sequences (ISs) [17], and these require sufficient sequence information flanking each repeat to determine if the sequence has recombined, or moved to a new location, resulting in a novel flanking sequence pairing. To date, studies have utilized split-read alignments from paired-end sequencing data as implemented in Pindel [18], an *A-Bruijn* graph-based algorithm as implemented in GRASPER [19], or optical mapping [20], to identify SVs in experimental bacterial populations. SV detection may also be achieved by using a mate-pair sequencing strategy with insert sizes that span the length of the repeat sequence, or with long sequence read technologies [21], but to our knowledge, such approaches have not yet been applied to MA studies [6, 22–24].

To date, the most comprehensive investigation into aerobic genome-wide mutation rate for *E*. *coli* estimated 1 × 10^−3^ mutations per genome per generation in a MA experiment on complex media, with mutation detection *via* whole genome re-sequencing, though this study did not measure SVs [6]. Furthermore, in an investigation into anaerobic spontaneous mutations [5], which was based on a single locus assay (*rpsL*), both a higher incidence of mutations and SVs for *E*. *coli* grown under anaerobic conditions, as compared to aerobic, was reported. It is unknown whether these trends are consistent on a genome-wide scale where the frequency of SVs may be influenced by the number and size of homologous sequences in the genome [25]. Here we report a comprehensive analysis of spontaneous mutations and the spectrum of mutational events in *E*. *coli* populations propagated under aerobic and anaerobic conditions. We show pronounced differences and thus shed light on the little studied effect of oxygen availability on evolutionary processes.

## Results

### Anaerobically grown *E*. *coli* REL4536 has a higher spontaneous mutation rate than aerobically grown *E*. *coli*

**Fig 1:**
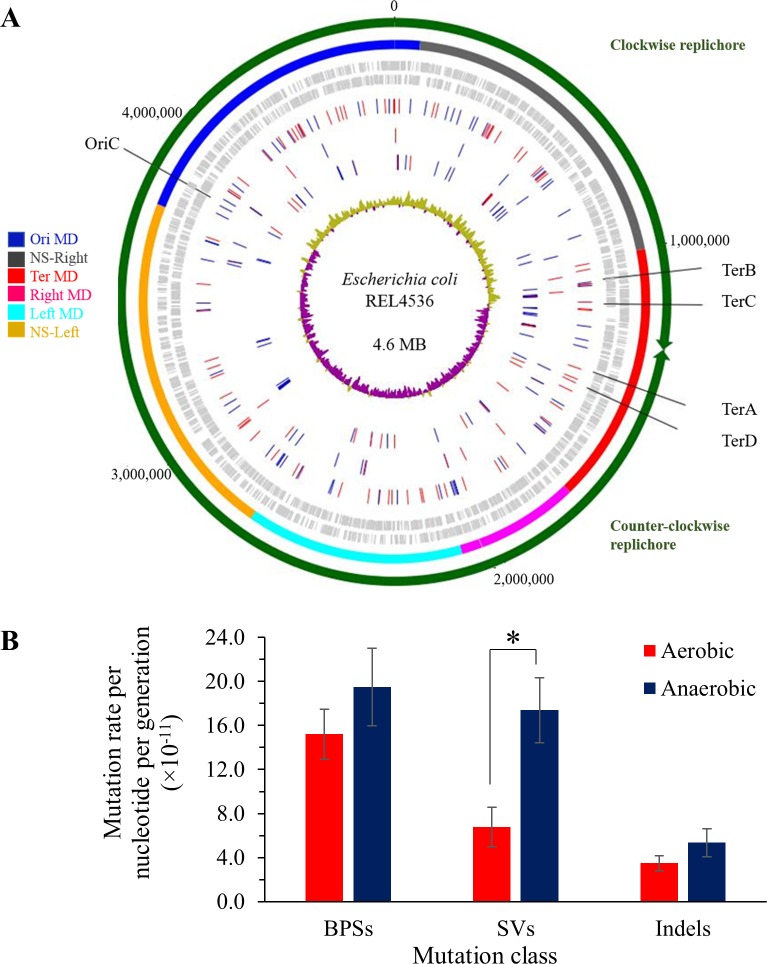
Mutation rates and spectra for aerobically and anaerobically grown REL4536. A) Genomic distribution of 124 and 158 mutations identified in the aerobically and anaerobically grown cells, respectively, mapped to the *E*. *coli* REL4536 genome. The outermost circle shows the genome organisation into replichores from the origin of replication, OriC, to the replication termination Ter sites. Arrows indicate the direction of replication for each replichore. The second circle shows the genome macrodomains (MDs), as defined previously [27, 28]. Coding sequences on the forward and reverse strands are shown on the third and fourth circles, respectively. BPSs, indels and SVs are shown on the fifth, sixth and seventh circles, respectively. Mutations in aerobic and anaerobic lineages are displayed in red and blue, respectively. The innermost circle displays the GC-skews, where green indicates an excess of G over C while purple indicates an excess of C over G. Detailed plots of cumulative mutation distribution against genome position are provided in S1 Fig) Mean mutation rates per nucleotide per generation of 24 aerobic and anaerobic lineages. Error bars are standard errors of the mean. * *p* < 0.05 by Mann-Whitney U-test.

*Escherichia coli* REL4536 has been aerobically adapted to a minimal glucose medium [15], and was used to establish MA lineages on minimal glucose agar plates. Fifty lineages per growth regime were propagated daily aerobically, and every three days anaerobically, for 4,500 and 3,456 generations respectively, representing 180 and 144 single colony bottlenecks, respectively. Mutations were detected in evolved lineages, 24 per treatment, through whole genome re-sequencing by directly mapping sequence reads to the reference sequence to detect small mutations, and *de-novo* assembly and alignment of contigs to the reference genome for detection of SVs (S1 Dataset). A total of 282 mutations were detected (Fig 1A), with 124 and 158 mutations identified in the aerobically and anaerobically grown cells, respectively (S2 Dataset). Of these, 147 mutations were BPSs (74 and 73 in the aerobically and anaerobically grown cells, respectively), 98 were SVs (33 and 65) and 37 were indels (17 and 20) (S2 Dataset). Based on these data, the estimated spontaneous mutation rate of 1.90 × 10^−3^ mutations per genome per generation for anaerobically grown cells was 1.7-fold greater [Mann-Whitney (MW) U = 196, *p* = 0.029] than that of the aerobically grown cells, at 1.15 × 10^−3^ mutations per genome per generation (Table 1). Despite differences in the bacterial strain and media used, and when SVs were excluded from the analysis, the aerobic mutation rate calculated in this study is within the range of previous genome-based estimates for *E*. *coli* [6] that do not account for SV mutations. Furthermore, independent estimates of mutation rates were assessed by Luria-Delbrück fluctuation assays [26] using a nalidixic acid marker (S1 Table). The mutation rates for anaerobically grown (2.34 × 10^−4^ mutations per genome per generation), as compared to aerobically grown (7.81 × 10^−5^ mutations per genome per generation) REL4536 were 3-fold greater (S1 Table), thus displaying the same trend of higher mutations rates for anaerobically grown cells. The 95% confidence limits for the two mutation rate estimates overlapped slightly, however (S1 Table). Furthermore, the lower mutation rate estimates observed by fluctuation assays, as compared to mutation accumulation with genome sequencing, highlight potential biases of the former method due to being based on single phenotypes. To assess whether the trend of higher anaerobic mutation rate was specific to REL4536, which had pre-adapted to aerobic growth over 10,000 generations, a comparison with REL4536’s non-aerobically adapted ancestor, REL606, was also performed. Similarly, higher mutation rates for anaerobically grown cells were observed (1.6-fold greater than aerobically grown, S1 Table), but again, the broad 95% confidence limits for these estimates overlapped slightly (S1 Table).

**Table 1:** Genome-wide spontaneous mutation rates for *E*. *coli* grown aerobically and anaerobically.

	No. of lines	No. of generations per bottleneck	No. of bottlenecks	Mutation rate per genome per generation ± SEM	Mutation rate per nucleotide per generation ± SEM
Aerobic	24	25	180	1.15 × 10^−3^ ± 1.46 × 10^−4^	2.55 × 10^−10^ ± 3.25 × 10^−11^
Anaerobic	24	24	144	1.90 × 10^−3^ ± 2.74 × 10^−4^	4.23 × 10^−10^ ± 6.07 × 10^−11^

### Minimal selection during the MA study

To investigate the influence of selection on the MA lineages, the positional and functional context of BPSs were examined (S2 Table). Given the codon usage of *E*. *coli* REL4536 genes and that the observed transition to transversion ratio was 2.08, the non-synonymous to synonymous substitution ratio (dN/dS) was 2.70 for aerobically grown cells, which is not significantly different from the expected ratio of 3.10 in the absence of selection (Pearson χ^2^ = 0.003, df = 1, *p* = 0.954). For anaerobically grown cells, given the transition to transversion ratio of 1.09 (S2 Table), the observed dN/dS of 1.63, was significantly different from the expected ratio of 3.05 (χ^2^ = 5.37, df = 1, *p* = 0.020). These results suggest that while all practicable steps were undertaken to minimize selection, the anaerobic MA lineages, which encountered a novel environment compared to their aerobic counterpart, were subject to some selection. As parallel BPS mutations were not encountered, selection in the MA study was considered to have been minimal. However, the observed dN/dS ratios in the aerobic and anaerobic environments imply that the anaerobically grown cells were subject to greater purifying selection than the aerobic ones.

The ratio of protein-coding to non-protein-coding sequences in *E*. *coli* REL4536 is 7.83. In this study, BPSs in coding versus non-coding regions (S2 Table) were present at a ratio of 4.29 for aerobically grown cells (χ^2^ = 4.25, df = 1, *p* = 0.04) and 2.17 for anaerobically grown cells (χ^2^ = 29.59, df = 1, *p* < 0.001). An elevated substitution rate in non-coding regions has been observed in other MA studies [6, 29, 30] and may be a general reflection of the preferential repair of BPS DNA damage in coding regions [6].

### BPS analyses

**Fig 2:**
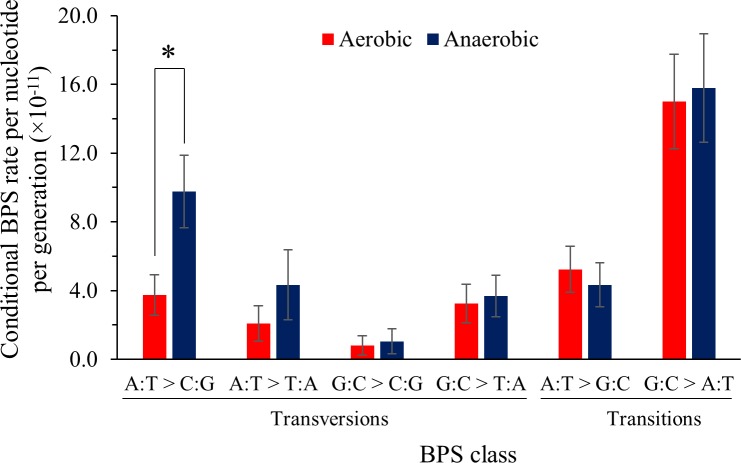
Conditional BPS mutation rates per generation in aerobically and anaerobically grown REL4536. Mutation rates for a total of 147 BPS mutations normalized to the number of A, T, G or C base pairs in each genome. Error bars are standard errors of the mean. * *p* < 0.05 by Mann-Whitney U-test.

**Fig 3:**
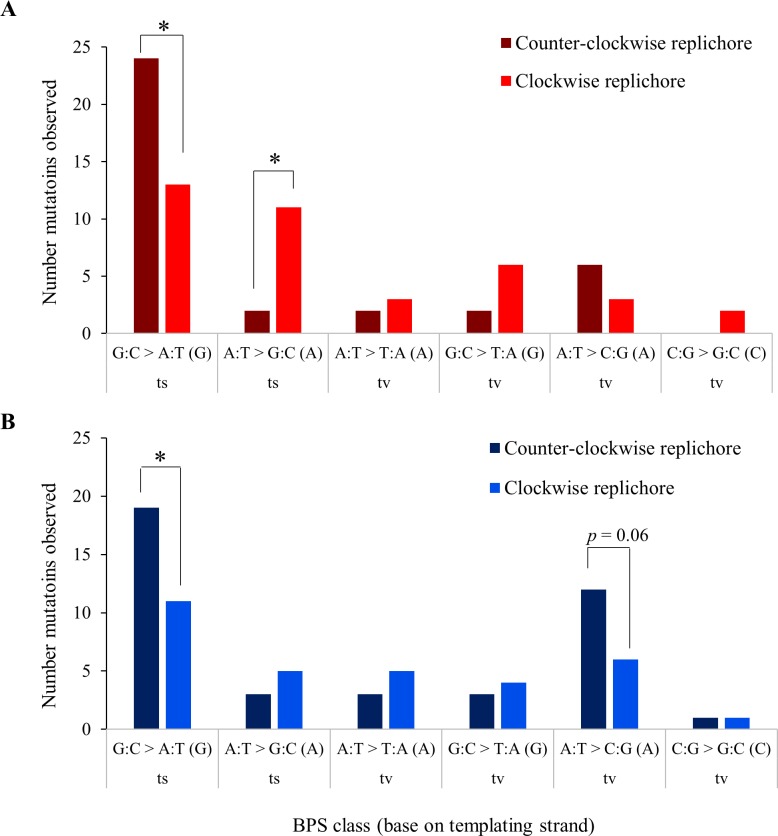
**Frequencies of BPSs in the clockwise and counter-clockwise replichores of A) aerobic and B) anaerobic lineages.** Expected values account for the uneven replichore sizes for REL4536 (clockwise replichore is 2.06 Mb, whereas the counterclockwise replichore is 2.54 Mb), and the assumption of equal mutation rates on the leading and lagging strand. * *p* < 0.05 by Pearson’s χ^2^-test.

BPSs were the most frequently observed mutation type, accounting for 52.1% of all mutations. A mean rate of 1.52 × 10^−10^ BPSs per nucleotide per generation was observed for the aerobically grown lines. This rate was not significantly different (*p* > 0.05, Student’s *t*-test) to the previously reported *E*. *coli* BPS rate of 1.99 × 10^−10^ BPSs per nucleotide per generation [6]. No significant differences between aerobic and anaerobic BPS rates were observed (Fig 1B). The predominant BPSs (comprising 45.6% of all BPSs) in both environments were G:C > A:T transitions (Fig 2). Substitution rates for A:T > C:G transitions were at least two-fold greater in anaerobically grown cells as compared to aerobically grown cells (MW U = 184, *p* = 0.017) (Fig 3).

A leading/lagging strand bias in the way BPSs accumulate has been observed in some MA studies [6, 31]. The two replichores of the *E*. *coli* chromosome start at the origin of replication and end at the terminus. During replication, the clockwise replichore strand, which templates lagging strand synthesis, is synthesized continuously while the counter-clockwise replichore strand, which templates the leading strand, is discontinuously synthesized [32]. Thus, any reciprocal mutational biases in leading and lagging strand synthesis will likely be reflected in the two replichores. To determine if there was DNA strand bias in this study, the number of BPSs per replichore were counted (Fig 3). REL4536 has unevenly sized replichores, where the clockwise replichore is 81% the size of the counterclockwise replichore due to an inversion, between two IS*1* elements, of a third of the genome [15]. When accounting for this size difference, A:T > G:C transitions were more frequent in aerobically grown cells when A templated the lagging strand rather than the leading strand (i.e. when A was in the conventionally reported 5' to 3' top strand of the reference) (χ^2^ = 4.54, *p* = 0.03). Similarly, G:C > A:T transitions were more frequent when G templated the leading strand rather than the lagging strand (χ^2^ = 6.07, *p* = 0.014), and this was also found for anaerobically grown cells (χ^2^ = 4.198, *p* = 0.04) (Fig 3B). Furthermore, A:T > C:G transversions also displayed a tendency toward strand bias in anaerobically grown cells (χ^2^-test, *p* = 0.06).

### SV and indel analyses

SVs comprised 34.8% of the mutations detected in this study (S2 Dataset). These were predominated by IS element mediated mutations (96%), though 2% of the SVs were mediated by other repeat-sequences, such as *rhs* elements. Two SVs were not associated with repeated sequences, suggesting a different mechanistic origin to IS element and repeat-mediated mutations. SVs were mainly detected in anaerobically grown cells, at a rate of 1.74 × 10^−10^ mutations per nucleotide per generation (Fig 1); 2.6-fold greater than that of aerobically grown cells (6.78 × 10^−11^ mutations per nucleotide per generation; MW U = 146.5, *p* = 0.002).

**Fig 4:**
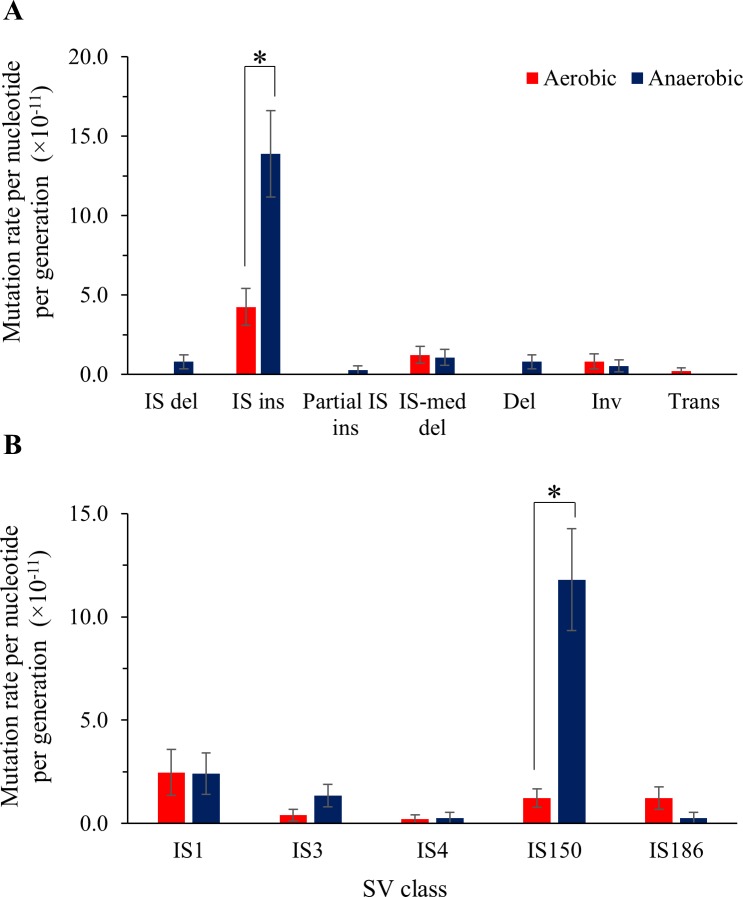
Mutation rates of SV classes in aerobically and anaerobically grown REL4536. Shown are mean mutation rates per nucleotide per generation for A) SV classes (del, deletion; ins, insertion; med, mediated; inv, inversion; and trans, translocation; and B) IS elements. Error bars represent standard errors of the mean. * *p* < 0.05 by Mann-Whitney U-test.

Analysis of the IS element-mediated mutations revealed that IS insertions were the most frequently observed type of SV (Fig 4). The aerobic IS insertion rate (4.52 × 10^−11^ mutations per nucleotide per generation) was similar to that previously reported [19]. Conversely, the rate of IS insertions for anaerobically grown cells was 3.1-fold greater than the aerobic rate at 1.39 × 10^−10^ mutations per nucleotide per generation (MW U = 155, *p* = 0.003; Fig 4A). For all other IS-mediated mutations (e.g. inversions and translocations), no significant differences between the mutation rates of aerobically and anaerobically grown cells were observed (*p* > 0.05, MW U-test; Fig 4A).

REL4536 possesses nine different IS element types (S3 Table) [33–36] and these differed in their mutation rates (Fig 4B). The mutation rates of SVs involving IS*1*, IS*3*, IS*4* and IS*186* did not differ between aerobic and anaerobically grown cells (Fig 4B), and mutations involving IS*2*, IS*30*, IS*600* or IS*911* were not detected. However, IS*150*-mediated mutations were the most abundant overall, and ~10-fold more insertions per generation were detected from anaerobically, as compared to aerobically, grown cells (MW U = 75, *p* < 0.001). A strong positive correlation between the copy number of each IS element type (S3 Table) and its aerobic mutation rate was observed (Pearson’s Correlation Coefficient R = 0.9187 and R^2^ = 0.844), however this was not the case for their anaerobic mutation rates (Pearson’s Correlation Coefficient R = 0.2326 and R^2^ = 0.0541) (S3 Table). This was mainly driven by the large difference in IS*150*-mediated mutations between the two environments. To further investigate why IS*150* was more active during anaerobic growth, consideration of IS element differences, their genomic contexts and expression levels were investigated and are discussed further below.

Indels accounted for 13.1% of all mutations identified in this study. The majority (51.4%) of indels were slippage events in regions of repetitive DNA (S2 Dataset). When data was directly compared to previous studies that reported indels of four nucleotides or less, a mean rate of 1.02 × 10^−4^ indels per nucleotide per generation was observed for the aerobically grown lines. This was similar to the previously reported rate of 8.34 × 10^−5^ indels per genome per generation [6] (*p* **>** 0.05, Student’s *t*-test). Additionally, rates of indels per generation were not significantly different in cells grown in the two environments (Fig 1B).

### Genomic distribution of mutations

To determine if mutations in the aerobically and anaerobically grown cells displayed notable trends in genomic distribution, the cumulative distributions of the BPSs, SVs and indels with regard to genome position were examined (S1 Fig). The cumulative distribution of SVs in both aerobically and anaerobically grown cells deviated from a linear relationship with chromosomal position, with a cluster of mutations occurring near the terminus of replication (S1C Fig). The relative abundance of BPSs, indels and SVs within the MDs, which are discrete, defined and structured regions of DNA that are unlikely to undergo recombination with each other due to functional constraints [27, 28] were examined (S4 Table). A chi-square test was used to determine whether classes of mutations were evenly distributed around the genome (S4 Table). BPSs were distributed evenly around the genome under both aerobic (χ^2^ = 2.67, df = 5, *p* > 0.05) and anaerobic (χ^2^ = 5.31, df = 5, *p* > 0.05) conditions (S4 Table). However, SVs did not occur at the relative frequencies expected if they arose evenly around the genome for both aerobic (χ^2^ = 29.47, df = 5, *p* < 0.001) and anaerobically (χ^2^ = 23.68, df = 5, *p* < 0.001) grown cells (S4 Table). Here, SVs are more prevalent within the terminus-containing Ter MD.

### Genes involved in recombination repair are more highly expressed under anaerobic conditions

Transcriptome analysis using RNAseq of *E*. *coli* REL4536 grown aerobically and anaerobically was undertaken to investigate the activities of various DNA replication and repair pathways involved in maintaining genome integrity, and to assess if their expression is consistent with observed patterns of mutation in the aerobic and anaerobic environments. In aerobically grown cells, 1,291 significantly up-regulated genes (Benjamini-Hochberg adjusted [BH adj.] *p* < 0.05) were identified; with 825 having at least two-fold greater expression than in anaerobically grown cells. In anaerobically grown cells, 1,245 significantly up-regulated genes were identified (BH adj. *p* < 0.05); with 747 genes having at least two-fold greater expression than in aerobically grown cells. Of the 147 genes known to be involved in DNA repair, transposition and replication in *E*. *coli*, 102 were significant differentially expressed (BH adj. *p* < 0.05) between the two environments with 30 up-regulated in aerobically grown cells and 72 up-regulated in anaerobically grown cells (S5 Table). The recombinational repair pathway [37, 38] displayed a significant difference in activity between the two environments, with many of the genes displaying greater expression under anaerobic conditions (S2 Fig and S5 Table). It is likely that the higher expression of genes involved in recombinational repair under anaerobic conditions is in response to the higher rate of SVs under these conditions.

The mismatch repair (MMR) pathway [37] also displayed differential activity between the two environments (S2 Fig and S5 Table). An essential repair system for the maintenance of genome fidelity [39, 40], a relative increase in the expression of genes involved in MMR under anaerobic conditions was observed. It is postulated that this may be in response to the higher rate of indels [39] and SVs [41, 42] or combinations thereof, under anaerobic conditions, though further investigation is required (S2 Fig and S5 Table).

Given the high rate of IS*150*-mediated mutations in anaerobically grown cells, IS element gene expression was of interest (S5 Table). IS*30*, IS*150* and IS*911* were the only IS elements with all genes displaying significantly greater expression (BH adj. *p* < 0.05) when anaerobically grown (S5 Table). No IS*30-*mediated mutations were detected in the MA experiment. IS*150* genes, *insJ* and *insK*, displayed 1.9 and 1.6-fold greater expression during anaerobic growth (*p* = 0.0001 and *p* = 0.0007, respectively), and IS*911* genes, *insN* and ECB_04146, showed similar expression increases during anaerobic cultivation, at 1.9 and 1.7-fold (*p* = 0.001 and *p* = 0.008, respectively), though the overall transcript abundance of IS*911* was approximately one third of that of IS*150* (S6 Table).

For each IS element type, copies had identical sequences, so it was not possible to determine the expression of any particular individual copy. To gain insight into whether there may be differences in expression of each IS*150* copy, we examined the genomic context of each IS*150* copy and its neighbouring genes (S7 Table). Consideration of expression level, orientation, and whether the IS element was located intergenically or intragenically, provided little indication as to why IS*150* activity was enhanced under anaerobic conditions. However, it is noted that one IS*150* copy disrupts *pflB* (which encodes pyruvate formate lyase) (S7 Table), and its deletion (and restoration of *pflB*) is predicted to enhance fitness under anaerobic growth by improving ATP yield during fermentation. We observed the deletion of IS*150* from *pflB* three times among the 24 anaerobic genomes sequenced, though these events contributed to only a small proportion of the 50 IS*150*-mediated mutations observed in the study overall. Insertion of IS*150* between *trg* and *mokB* appears to be a hotspot, where it was observed twice amongst the aerobic genomes sequenced, and eight times amongst the anaerobic, as well as in previous studies [15]. Why the frequency had increased under anaerobic growth is unclear.

## Discussion

Genome-wide analyses of experimental MA lineages revealed spontaneous mutation rates of 2.55 × 10^−10^ and 4.23 × 10^−10^ mutations per nucleotide per generation for aerobically and anaerobically grown *E*. *coli*, respectively. These rates are greater than those obtained *via* fluctuation assays, consistent with trends found in other MA studies [6, 43]. The difference between the anaerobic and aerobic mutation rates (1.7-fold) is consistent with findings based on a single locus assay, where anaerobically grown cells of a different *E*. *coli* strain also displayed a mutation frequency that was almost two-fold greater than that of aerobically grown cells [5]. While the number of mutations obtained in this study (282) was considerably lower than that for other MA-based studies that employed the use of mutator strains [6, 31, 44], the use of wild-type strains was necessary to accurately measure spontaneous mutation rates and spectra without biases mediated by the mutator, depending on its mode of action. The analysis of additional clones from independent MA lineages, and propagation for longer periods of time will further increase the power of this approach. However, our data expand on findings that SVs are more prevalent in anaerobically grown cells [5], and we provide detailed insight into the genetic elements that predominantly contribute to SVs, their genomic distribution and impact on genome re-organization.

Differences in mutation rates may be due to differences in growth rates and timings of cell cycle events in aerobically and anaerobically grown cells. As faster growing cells spend a relatively larger proportion of the cell cycle in S phase [45, 46] it is predicted that aerobically grown cells may be more vulnerable to mutations that predominantly arise during DNA synthesis [47], where DNA is particularly susceptible to damage as it transiently exists in single-stranded form [6, 48]. In contrast, mutations that arise independently of DNA synthesis can be identified by assessing mutation rates expressed per unit time (rather than generation). In this manner, mutation types that occur independently of aerobic status and mutations specific for growth under aerobic and anaerobic environments can be identified (S3 Fig and S4 Fig). For example, mutation rate estimates, calculated per generation, for A:T > C:G transversions were greater under anaerobic conditions. However, when considered per day, the mutation rates were equivalent under aerobic and anaerobic conditions. This suggests that these mutations occur at a temporal rate that is independent of the cell-cycle, and that there is no net impact on the rates of these mutations due to cellular physiology. In contrast, G:C > A:T transitions were observed at equivalent rates per generation, and greater under aerobic conditions when calculated per day (S3 Fig). This is consistent with these BPSs predominantly occurring during DNA replication, where single-stranded DNA is particularly prone to cytosine deamination [49]. Mutation rate estimates for IS*150* elements were generally greater under anaerobic conditions when calculated both per generation and per unit time (S4 Fig). Thus, these IS*150* insertions were likely the result of the net impact of cellular physiology.

In this study, transitions were frequently observed, consistent with a typical mutation bias that comes with increasing A:T content of bacterial genomes [50–52], though it is noted that some recent MA studies observed mutational biases toward increasing G:C content [30, 43]. Asymmetries in the rates of transitions were also observed in both aerobically and anaerobically grown cells, which demonstrates the impact that physiological conditions have on specific mutation types. In aerobically grown cells, A:T > G:C were significantly more abundant when A templated the lagging strand rather than the leading strand, consistent with previous findings [6]. Furthermore, G:C > A:T transitions were more prevalent when G templated the leading strand rather than the lagging strand in both aerobically and anaerobically grown cells. The underlying mechanisms driving the bias are not understood but the semi-discontinuous nature of DNA replication could create a mutation strand bias [32, 53] for mutations that arise more frequently when DNA is in single-stranded form. The complementary strand of the template leading strand is synthesized discontinuously *via* Okazaki fragments, thus the template leading strand is in a single-stranded state for a relatively longer time period than the template lagging strand, whose complementary leading strand is synthesized continuously. Mutation asymmetry will result if there is bias for mutations that predominate on the leading or lagging strands [54–56], together with differential replichore sizes and nucleotide compositions, as is the case for *E*. *coli* REL4536 (S8 Table). Spontaneous deamination of cytosine and adenine are frequently occurring DNA lesions known to cause C:G > T:A and A:T > G:C transitions, respectively [37]. As single-stranded DNA is more prone to cytosine and adenine deamination [48, 49, 57], it is expected that C:G > T:A and A:T > G:C transitions would be more abundant when C or A template the lagging strand. These mutation patterns were observed in the anaerobically grown-cells and aerobically grown cells, respectively. In addition, mutational strand bias may also result from transcription bias (S8 Table), where the non-transcribed strand is repaired more efficiently than the transcribed strand [32, 53, 58]. The observed strand biases may also result directly from the different physiological conditions associated with aerobic and anaerobic growth, which may affect the rate at which specific BPSs arise in single-stranded DNA. Acidic fermentation end-products lower pH and can directly cause DNA damage [59] or induce mutagenic cellular activities [9, 60]. Depurination of DNA takes place to a significant extent under *in vivo* physiological conditions, however it occurs more frequently with decreasing pH [61]. During replication, a random base is incorporated opposite “blank” apurinic sites, likely resulting in a BPS. Depurinated DNA is repaired by the base excision repair (BER) pathway, and nearly half of the genes associated with this pathway were significantly up-regulated in cells grown under anaerobic conditions (S5 Table). BER is predicted to be active during both anaerobic and aerobic growth, where under anaerobic conditions, activity would be largely in response to acid-induced depurination, while under aerobic conditions, BER is the primary pathway for the repair of ROS-induced DNA damage repair [37]. In *E*. *coli*, arginine and anaerobiosis are required for the induction and function of acid resistance system 3 [62–64]. Genes involved in arginine synthesis and DNA replication, repair and transposition were significantly up-regulated in anaerobically grown cells (S2 Fig and S9 Table), suggesting that cells experience acid stress, and this contributes to the higher mutation rates observed. Exogenous agents, or redox properties of DNA, which result in the generation of mutagenic tautomers of nucleobases [65, 66], could potentially also contribute to the higher mutation rate and spectrum observed under anaerobic conditions.

Recent MA studies have observed that local nucleotide context can affect site-specific mutation rate [29, 31, 67]. For example, higher mutation rates were observed at nucleotides that were flanked, on either or both sides, by a C:G base pair in different prokaryotes [29, 31], most likely due to base stacking [68]. As we have observed that chromosomal position and leading/lagging strand synthesis can affect mutation rate differences between the two environments, it is possible that neighboring nucleotide context could also drive the mutational differences observed in this study.

The elevated level of IS element movement displayed in the anaerobic lineages, particularly for IS*150* (Fig 4), suggests that IS elements play a considerable role in generating genetic diversity, which may contribute to adaptation during growth in anaerobic environments. In previous studies, IS elements have been shown to generate beneficial mutations that increase organismal fitness [69], while other studies have shown that IS element-mediated mutations can occur in response to starvation and other cellular stresses [70]. In the present study, a number of SVs that arose in the anaerobically grown MA lineages, including IS*150* deletion from *pflB*, which is predicted to improve energy yield during fermentation, appear to have been selected for in a closely related study of the adaptation of *E*. *coli* REL4536 to the anaerobic environment [71]. Though IS element transposition can be regulated in various ways [34, 35], the factors that enhance IS element activity under anaerobic conditions remain unknown. A recent MA study investigating IS element movement in various *E*. *coli* strains rarely detected IS*150* transposition [72] while IS*150* was found to be highly active during aerobic long-term evolution experiments that were initiated with the ancestral strain to REL4536 [20, 69, 73]. This suggests that the increased IS*150* activity in this study may be strain-specific, where REL4536 has a large number of IS*150* target sites. Examination of IS element gene expression under aerobic and anaerobic growth revealed that IS*1*, and not IS*150*, displayed the highest levels of gene expression, and copies of IS*1* were over four times more abundant than that of IS*150* within the REL4536 genome. The present study examined transcriptome activity only at early stationary phase, and more comprehensive analysis of the transcriptome dynamics throughout culture growth (e.g. during log phase and late stationary phase) may reveal differences in IS expression activity that better correlate with the observed mutations rates. However, post-transcriptional regulatory factors may also contribute to the apparently higher activity of IS*150*. IS*3* family elements (which include IS*150*) use a non-standard mechanism of translation, programmed translational frameshifting (PTF) [33, 74], to generate transposases from their two consecutive, out-of-phase, genes. The impact of anaerobic cellular physiology on this process is not known, and further investigation into these factors may provide insight into the higher levels of IS*150* activity in anaerobically grown cells. Furthermore, IS element transposition is influenced by the relative activities of the InsA and InsB protein homologs [75], and the *ins* gene homologs differed in their relative expression for the IS elements in REL4536, and under aerobic and anaerobic conditions. Further post-transcriptional insight into IS element activities, and their contributions to the observed mutation rates, may be obtained by determining the abundance and activities of transposases for each IS element type during aerobic and anaerobic growth.

It is further noted that the elevated mutation rate of IS*150* may be due to a subset of cells within the population, or from activities of particular IS*150* copies, arising from their genomic context. To investigate these possibilities further, a single-cell monitoring approach, or the use of gene reporters specific to each IS*150* copy, would be informative. SVs prevailed around the chromosome terminus, which is consistent with previous reports [76], and likely due to structural constraints to either maintain the genome symmetry between the origin and terminus of replication, or to maintain genome organization [77].

Overall, the findings of this study provide new insight into the mutagenic and physiological pressures associated with aerobic and anaerobic growth, and provide a framework upon which the evolution of facultative anaerobes can be based.

## Materials and Methods

### MA lineages

*Escherichia coli* REL606 and REL4536, a 10,000^th^ generation descendent of REL606 from the *E*. *coli* long-term evolution experiment [15], was kindly provided by Richard Lenski (Michigan State University). MA lineages of *E*. *coli* REL4536 were grown on 1.5% Davis minimal (DM) agar plates [78] supplemented with 0.02% (w/v) glucose, 0.01% (w/v) magnesium sulphate and 0.0002% (w/v) thiamine. Single colonies from aerobic MA lineages were re-streaked every 24 h for 180 bottlenecks, and every 72 h for 144 bottlenecks for anaerobic lineages, and grown at 37°C. MA lineages were routinely checked for contamination every 14 bottlenecks. Phage tests [14] were conducted using representative colonies from each lineage where sensitivity to Coliphage T5 and resistance to Coliphage T6 indicated the correct strain of *E*. *coli* was present. MA lineages were frozen every 15 bottlenecks by suspending the streaked colony in 400 μL of 15% glycerol and storage at -85°C.

To initiate the MA lineages, the ancestral *E*. *coli* REL4536 strain was streaked on a DM agar plate and incubated at 37°C. After 24 h, 100 colonies were randomly selected and each colony initiated an independent MA line by streaking onto aerobic or anaerobically prepared DM plates. Agar plates were divided into eight sectors to allow eight separate lineages to be propagated in parallel per agar plate. Prior to streaking a new plate, dots were randomly marked on the base of the plate in each sector. Thus for the next transfer, the colony closest to the mark was selected for propagation, ensuring that there was no bias towards colonies of a particular size or phenotype. Aerobic MA lineages were propagated in a Class II Type a/B3 biohazard cabinet (NuAire) while anaerobic MA lineages were propagated in an anaerobic glove box (Coy Laboratory Products Inc.) containing a 92% CO_2_:8% H_2_ atmosphere, and placed in AnaeroJar canisters (Oxoid) to maintain an anaerobic atmosphere after removal from the chamber.

The number of cell divisions during the time period between bottlenecks was estimated by counting the average number of cells in 10 colonies of *E*. *coli* REL4536 after 24 h in the aerobic environment and the average number of cells in 10 colonies of *E*. *coli* REL4536 after 72 h in the anaerobic environment. The number of cell divisions, or generations, of colony growth were calculated with the equation *G* = (*logN*)/*log2*, where *G* is the number of generations and *N* is the final number of cells in a colony. This yielded an estimate of approximately 25 and 24 generations for the aerobic and anaerobic environment, respectively.

### Fluctuation assays

Mutation rates for spontaneous nalidixic acid resistance (Nal^R^) were estimated by fluctuation assays using aerobic and anaerobic DM2000 media [79]. In each environment, 60 cultures in 0.2 mL DM media supplemented with 0.20% (w/v) glucose, 0.01% (w/v) magnesium sulphate and 0.0002% (w/v) thiamine (DM2000) were inoculated with a 10^−6^ dilution of a stationary phase culture of the appropriate strain. Cultures were grown until stationary phase at 37°C with shaking, after which they were plated on solid medium containing the selection agent. Plates were incubated at 37°C for 48–72 h, after which, colonies were counted to determine mutation rates. To assess for nalidixic acid resistance, cultures were plated on LB agar plates supplemented with 30 μg/mL nalidixic acid (n = 48) and on LB agar plates with no antibiotic (n = 12). Mutation rates were calculated using the Ma-Sandri-Sarkar Maximum Likelihood Estimator (MSS-MLE) method [80], as implemented in FALCOR [81]. Estimates of mutation rates per nucleotide were obtained by normalizing the obtained mutation rate per locus estimates to the number of nucleotides that are assumed to be responsible for conferring the mutant phenotype, as implemented by Lee et al. [6]. Resistance to nalidixic acid may be conferred by 18 different point mutations in the *gyrA* gene, and 2 point mutations in the *gyrB* gene [82–84].

### DNA sequencing

High-quality genomic DNA was extracted from cultures grown in lysogeny broth using a phenol-based extraction method [85] and ethanol precipitation. To prepare DNA for whole genome sequencing, 100–250 mL of overnight LB cultures were used. For each lineage, a culture was inoculated from a randomly chosen colony after the lineages had been maintained for the desired number of bottlenecks. Aerobic cultures were grown in serum bottles (Wheaton Science Products) in media prepared aerobically and covered with AeraSeal Breathable Films. All anaerobic work was carried out in an anaerobic glove box and anaerobic cultures were grown in serum bottles in media prepared anaerobically and sealed with butyl rubber stoppers. All cultures were incubated at 37°C with an orbital shaking of 150 RPM for 24 h. Cells were harvested by centrifugation at 8,000 *g* for 10 min at 4°C in 500 mL centrifuge bottles. Media was discarded and cell pellets were snap-frozen in liquid nitrogen and stored at -20°C. For cell lysis, frozen pellets were re-suspended in 1/20^th^ culture volume lysis buffer [30 mM NaCl, 50 mM pH 8.0 Tris-HCl, 5 mM pH 8.0 EDTA and 10% (w/v) SDS] and the solutions were transferred to 15 mL Falcon tubes (Becton-Dickinson). Cells were once again harvested by centrifugation at 8,000 *g* for 10 min at 4°C. Supernatant was discarded and cells were re-suspended in 1/50^th^ volume lysis buffer containing 20 mg/mL lysozyme (Sigma-Aldrich) and 10 μg/mL RNase A (Sigma-Aldrich). Samples were incubated at 37°C with orbital shaking at 150 RPM for 2 h to allow cell lysis. EDTA/SDS solution (0.25 M EDTA and 20% SDS), 2 mL, was added to each sample and samples were incubated for another 2 h at 65°C. Finally, 100 μL of 20 mg/mL Proteinase K (Sigma-Aldrich) was added to each sample and the mixture was incubated overnight at 65°C. For all extractions, UltraPure phenol:chloroform:isoamyl alcohol (25:24:1, v:v:v; Invitrogen) and chloroform:isoamyl alcohol (24:1, v:v; Sigma-Aldrich) were used. DNA was quantified using the Quant-iT dsDNA Broad-range (BR) Assay kit (Life Technologies) and measured on a Qubit 2.0 fluorometer (Life Technologies) as per the manufacturer’s instructions. DNA purity and quality was assessed using a NanoDrop Technologies ND-1000 UV-Vis Spectrophotometer.

As it had previously been implicated that SVs are prevalent in anaerobically grown cells [5] it was vital to be able to accurately identify these mutations. SVs are often mediated by large repetitive sequences [17], thus sequence pair information and library insert size are critical for detecting them using *de novo* assemblies, as this enables unique reads that flank repeated sequences to be linked. Within the REL4536 reference genome, IS*150* was the largest IS element at a length of 1446 bp. Therefore, to ensure that all IS element meditated SV mutations could be detected, DNA was sequenced with 2 kb mate-pair inserts on the Illumina HiSeq 2000 platform (BGI). Paired-end sequencing with 90 bp reads resulted in over 98% genome coverage with the mean read depth ± standard deviation was 127.6 ± 11.1 and 64.0 ± 24.8 for the aerobic, and anaerobic genomes, respectively (S1 Dataset). The DNA sequences for the MA lines are deposited in the National Center for Biotechnology Information (NCBI) Sequence Read Archive, BioProject PRJNA315479.

### Mutation detection

The *E*. *coli* REL4536 reference genome was manually annotated using information provided by Barrick et al. [15] and the *E*. *coli* REL606 GenBank file (NCBI Reference Sequence NC_012967.1). Sequence data from BGI had been filtered to remove reads containing ≥ 10% unreadable bases, ≥ 20% low quality (≤ Q20) bases, adapter contamination or duplicate read-pairs and FastQC [86] was used to check the quality of the sequence data before analysis. BPSs, indels and MGE movement were identified using breseq [87, 88] with default parameters. To detect SVs, genomes were assembled *de novo* using SPAdes 3.0.0 [89] with default parameters. Assemblies were aligned to the reference *E*. *coli* REL4536 genome using the NUCmer pipeline in MUMmer [90] and Mauve [91] with default parameters to identify SV breakpoints. Verification of SV breakpoints was carried out using PCR using primers in S10 Table. PCR (25 μL total reaction volume) was performed using 1× reaction buffer (Invitrogen), 2 mM Mg^+2^ (Invitrogen), 0.2 mM deoxyribonucleotide triphosphate mixture (Invitrogen), 0.2 μM forward primer, 0.2 μM reverse primer, 0.02 unit/μL of Platinum High Fidelity Taq DNA polymerase (Invitrogen) and 1–100 ng of genomic DNA. PCR amplifications were performed on Eppendorf proS Mastercycler PCR machines and a standard PCR programme was used: initial denaturation at 94°C for 3 min, followed by 30 cycles of 94°C for 30 sec, annealing temperature (primer dependent) for 30 sec and extension at 68°C for 1 min per kb of product. The final elongation was for 10 min at 68°C. Using UltraPure Agarose (Invitrogen), 1% (w/v) gels were made up in 1× Tris-acetate-EDTA (TAE) buffer containing 1× SYBR safe nucleic acid dye (Life Technologies) to separate and visualize the PCR products.

In this study, an increase in IS element copy number, as well as a change in the location of an IS element, were both counted as IS insertions. Spontaneous mutation rates for aerobically and anaerobically grown *E*. *coli* lineages were calculated with the equation μ = *m*/(L*N*T), where μ is the mutation rate per genome per generation, *m* is the total number of observed mutations, *L* is the number of MA lineages, *N* is the number of generations between bottlenecks and *T* is the number of bottlenecks (180 for aerobic lineages and 144 for anaerobic lineages). To obtain the mutation rate per nucleotide per generation, the equation μ/*G* was used, where *G* is the size of the genome that was sequenced (S1 Dataset). To obtain the mutation rate per genome per day, the equation μ*'* = *m*/(L*D) was used, where μ*'* is the mutation rate per genome per day, *m* is the total number of observed mutations, *L* is the number of MA lineages and *D* is the number of days of evolution (180 for aerobic lineages and 432 for anaerobic lineages).

### Differential gene expression

The transcriptomes of three aerobic and two anaerobic stationary phase *E*. *coli* REL4536 cultures were sequenced. Aerobic cultures were grown in 250 mL serum bottles for 9 h while anaerobic cultures were grown for 16 h. All cultures were grown in 50 mL DM25 aliquots at 37°C. Cells were harvested by centrifugation at 8,000 *g* for 10 min at 4°C. Media was discarded and cell pellets were snap-frozen in liquid nitrogen. RNA was extracted from cultures using a hot lysis buffer and acid phenol-based extraction method [85] and isopropanol precipitation. Samples were lysed with 5 mL of 2% SDS solution at 65°C and poured into 10 mL of phenol:chloroform:isoamyl alcohol (125:24:1, v:v:v, pH 4.5; Ambion) and incubated at 65°C for 15 min. Following RNA extraction, Turbo DNase (Ambion) was used to treat the samples as per manufacturer’s instructions. Each sample was split into five 20 μL aliquots and two rounds of DNase treatment were performed on each aliquot. Following DNase treatment, RNA samples were purified using the Qiagen RNeasy Mini kit as per the manufacturer’s instructions. For each sample, the DNase-treated aliquots were pooled together before purification. RNA quality was measured using the Agilent 2100 Bioanalyzer (Agilent Technologies) with the RNA 6000 Nano Chip kit according to the manufacturer’s instructions while RNA was quantified using the Quant-iT RNA Assay kit (Life Technologies) and measured on a Qubit 2.0 fluorometer.

Total RNA libraries of 200 bp inserts were sequenced by Illumina HiSeq 2000 at BGI. Paired-end sequencing with 90 bp reads was performed. The quality of reads was assessed by using FastQC [86] and any reads with a quality score ≤ Q28 were trimmed using Trim Galore![92]. Bowtie 2 [93] was used with default parameters, to remove any sequence reads aligning to ribosomal RNA, transfer RNA and non-coding RNA sequences. EDGE-pro (Estimated Degree of Gene Expression in PROkaryotes) [94] and DESeq2 [95] were used on the remaining reads to identify differential expression between aerobic and anaerobic environments. The RNA sequence data is available at NCBI GEO accession GSE80451.

### Statistical analysis

Standard statistical tests were performed in GenStat 17^th^ Edition.

## Supporting Information

S1 Dataset Genome re-sequencing reference-based mapping and *de novo* assembly statistics for MA clones. (XLSX)

S2 Dataset Mutations detected amongst the aerobic and anaerobic lineages. (XLSX)

S1 Table *E*. *coli* mutation rates calculated from fluctuation assays. (DOCX)

S2 Table Spectra of BPSs amongst the aerobic and anaerobic lineages. (DOCX)

S3 Table REL4536 IS element features and mutation rates. (DOCX)

S4 Table Relative distribution of mutations within the macrodomains of *E*. *coli*. (DOCX)

S5 Table Expression values of *E*. *coli* REL4536 genes involved in DNA repair, replication and transposition. (DOCX)

S6 Table Relative abundance of IS gene transcripts in aerobic and anaerobically grown cells. (DOCX)

S7 Table IS*150* genome location. (DOCX)

S8 Table Clockwise and counter-clockwise replichores of *E*. *coli* REL4536. (DOCX)

S9 Table Expression of genes involved in arginine biosynthesis. (DOCX)

S10 Table Primers used to verify SV break points. (DOCX)

S1 Fig Cumulative distribution of mutations in the genomes of aerobic and anaerobic MA lineages. Shown are the relationships between cumulative mutations and chromosomal position for A) expected random distribution of mutations around the genome, B) BPSs, C) SVs, and D) indels.
(TIF)

S2 Fig Significantly up-regulated genes, as a proportion of genes in the pathway, known to be involved in DNA repair and replication (classified by pathway). To identify significant expression, Benjamini-Hochberg adjusted *p* < 0.05 were used. Asterisk denotes a significant enrichment of the gene list under anaerobic conditions by Fisher’s exact test (*, *p* < 0.05).
(TIF)

S3 Fig Comparison of mutation rates expressed per generation and per unit time. Shown are: Mean mutation rates of mutational classes expressed A) per nucleotide per generation, B) per nucleotide per day of growth, C) Conditional BPS rates expressed per nucleotide per generation and D) Conditional BPS rates expressed per nucleotide per day of growth. Error bars represent standard errors of the mean. * denotes *p* < 0.05 by Mann-Whitney U-test.
(TIF)

S4 Fig Comparison of SV mutation rates. Shown are: A) and C) Mutation rates of different SV classes expressed per nucleotide per generation, and B) and D) Mutation rates of different SV classes expressed per nucleotide per day of growth. Error bars represent standard errors of the mean. * denotes *p* < 0.05 by Mann-Whitney U-test.
(TIF)
