# Supplementary material for: Anaerobically Grown Escherichia coli Has an Enhanced Mutation Rate and Distinct Mutational Spectra
Source: PLoS Genet. 2017 Jan 19;13(1):e1006570. doi: 10.1371/journal.pgen.1006570 (PMC5289635; doi:10.1371/journal.pgen.1006570)
Supplement: S1 Table — (DOCX) [file pgen.1006570.s003.docx]

**S1 Table.** ***E. coli* mutation rates calculated from fluctuation assays.**

| Strain | Environment | Mutation rate per locus  per generation  (× 10^-9^)^†^ | 95% CL^††^  (× 10^-9^) | Mutation rate per genome  per generation  (× 10^-4^) | 95% CL  (× 10^-4^) |
| --- | --- | --- | --- | --- | --- |
| REL4536 |  |  |  |  |  |
|  | Aerobic | 0.34 | 0.13 – 0.62 | 0.78 | 0.30 – 1.42 |
|  | Anaerobic | 1.02 | 0.45 – 1.75 | 2.34 | 1.03 – 4.02 |
| REL606 |  |  |  |  |  |
|  | Aerobic | 0.57 | 0.26 – 0.95 | 1.31 | 0.60 – 2.18 |
|  | Anaerobic | 0.92 | 0.41 – 1.58 | 2.11 | 0.94 – 3.63 |

^†^The single mutation rate estimate per treatment was calculated in FALCOR from 60 independent cultures with assessment by spontaneous nalidixic acid resistance.

^††^95% confidence limit of the mutation rate estimate was generated by FALCOR.
