# Supplementary material for: Anaerobically Grown Escherichia coli Has an Enhanced Mutation Rate and Distinct Mutational Spectra
Source: PLoS Genet. 2017 Jan 19;13(1):e1006570. doi: 10.1371/journal.pgen.1006570 (PMC5289635; doi:10.1371/journal.pgen.1006570)
Supplement: S2 Table — (DOCX) [file pgen.1006570.s004.docx]

S2 Table. Spectra of BPSs amongst the aerobic and anaerobic lineages.

|  |  | Aerobic | Anaerobic |
| --- | --- | --- | --- |
| *Substitution type* | | | |
| Transitions |  | 50 | 38 |
|  | A 🡪 G | 10 | 6 |
|  | T 🡪 C | 3 | 2 |
|  | G 🡪 A | 18 | 17 |
|  | C 🡪 T | 19 | 13 |
| Transversions |  | 24 | 35 |
|  | A 🡪 C | 4 | 9 |
|  | A 🡪 T | 1 | 4 |
|  | C 🡪 A | 1 | 6 |
|  | C 🡪 G | 0 | 1 |
|  | G 🡪 C | 2 | 1 |
|  | G 🡪 T | 7 | 1 |
|  | T 🡪 A | 4 | 4 |
|  | T 🡪 G | 5 | 9 |
| *Region of substitution* | | | |
| Coding sequences | | 60 | 50 |
|  | Synonymous | 16 | 19 |
|  | Non-synonymous | 44 | 31 |
| Non-coding sequences | | 14 | 23 |
