# Supplementary material for: Anaerobically Grown Escherichia coli Has an Enhanced Mutation Rate and Distinct Mutational Spectra
Source: PLoS Genet. 2017 Jan 19;13(1):e1006570. doi: 10.1371/journal.pgen.1006570 (PMC5289635; doi:10.1371/journal.pgen.1006570)
Supplement: S3 Table — (DOCX) [file pgen.1006570.s005.docx]

**S3 Table. REL4536 IS element features and mutation rates.**

| Element | Copies | Size (bp) | Genes | IS family | Mechanism^†^ | Target length (bp) | Aerobic mutation rate (per site per generation, ×10^-11^)^††^ | Anaerobic mutation rate (per site per generation, ×10^-11^)^††^ |
| --- | --- | --- | --- | --- | --- | --- | --- | --- |
| IS1 | 26 | 777 | *insA, insB** | IS1 | CoPa | 8-9 | 2.47 ± 1.11 | 2.41 ± 1.01 |
| IS2 | 1 | 1,128 | *insC, insD*** | IS3 | CoPa | 5 | 0 | 0 |
| IS3 | 5 | 1,260 | *insE, insF*** | IS3 | CoPa | 3-4 | 0.41 ± 0.28 | 1.34 ± 0.54 |
| IS4 | 1 | 1,438 | *insG* | IS4 | CuPa | 10-13 | 0.21 ± 0.21 | 0.27 ± 0.27 |
| IS30 | 1 | 1,187 | *insI* | IS30 | CoPa | 2-3 | 0 | 0 |
| IS150 | 6 | 1,446 | *insJ, insK*** | IS3 | CoPa | 3-4 | 1.23 ± 0.45 | 11.80 ± 2.46 |
| IS186 | 5 | 1,349 | *insL* | IS4 | n/a | 10-11 | 1.23 ± 0.54 | 0.27 ± 0.27 |
| IS600 | 1 | 1,180 | *yis1, yis2*** | IS3 | n/a | 3 | 0 | 0 |
| IS911 | 1 | 1,198 | *insN, ECB_04146*** | IS3 | CoPa | 3-4 | 0 | 0 |
|  | | | |  |  |  |  |  |
| Correlation with copy number, Spearman’s Rho R (*P*-value) | | | | |  |  | 0.939 (0.0002) | 0.900 (0.0009) |
| Correlation with copy number, Pearson’s Correlation Coefficient R (R^2^) | | | | | |  | 0.919 (0.844) | 0.233 (0.054) |

^†^Where known, mechanism of acitivity is listed: CoPa (copy and paste), CuPa (cut and paste) and n/a (not available).

^††^Mean mutation rate ± standard error of the mean is given.

^*^Recoding (i.e. -1 translational frameshift at A_AA.A_AA.C) is used to generate the functional transposase encoded by the two genes.

^**^Recoding (i.e. -1 translational frameshift at A_AA.A_AA.G) is used to generate the functional transposase encoded by the two genes.
