# Supplementary material for: Anaerobically Grown Escherichia coli Has an Enhanced Mutation Rate and Distinct Mutational Spectra
Source: PLoS Genet. 2017 Jan 19;13(1):e1006570. doi: 10.1371/journal.pgen.1006570 (PMC5289635; doi:10.1371/journal.pgen.1006570)
Supplement: S4 Table — (DOCX) [file pgen.1006570.s006.docx]

S4 Table. Relative distribution of mutations within the macrodomains of *E. coli*.

|  | Ori MD | NS^Right^ | Ter MD | Right MD | Left MD | NS^Left^ | Pearson’s Chi-square^†^  (df = 5) | |
| --- | --- | --- | --- | --- | --- | --- | --- | --- |
| *Aerobic proportion of mutations* | | | | | | | |  |
| BPSs | 0.26 | 0.18 | 0.19 | 0.07 | 0.15 | 0.16 | χ^2^ = 2.67, *p* = 0.751 | |
| SVs | 0.15 | 0.15 | 0.48 | 0.06 | 0.15 | 0.00 | χ^2^ = 29.47, *p* < 0.001 | |
| Indels | 0.23 | 0.00 | 0.23 | 0.23 | 0.08 | 0.23 | χ^2^ = 10.31, *p* = 0.067^††^ | |
| *Anaerobic proportion of mutations* | | | | | | | |  |
| BPSs | 0.22 | 0.21 | 0.14 | 0.14 | 0.08 | 0.22 | χ^2^ = 5.31, *p* = 0.379 | |
| SVs | 0.15 | 0.15 | 0.48 | 0.06 | 0.15 | 0.00 | χ^2^ = 23.68, *p* < 0.001 | |
| Indels | 0.16 | 0.21 | 0.16 | 0.05 | 0.16 | 0.26 | χ^2^ = 0.63, *p* = 0.987^††^ | |
| *Expected proportion of mutations for even distribution across the genome* | | | | | | | |  |
| BPSs | 0.24 | 0.19 | 0.16 | 0.10 | 0.12 | 0.19 |  | |
| GCRs | 0.18 | 0.17 | 0.41 | 0.07 | 0.09 | 0.07 |  | |
| Indels | 0.19 | 0.13 | 0.19 | 0.13 | 0.13 | 0.25 |  | |

^†^Null hypothesis is that mutations are distributed evenly across the genome.

^††^The criteria for the statistical test was not met as expected cell count was < 5.
