# Supplementary material for: Anaerobically Grown Escherichia coli Has an Enhanced Mutation Rate and Distinct Mutational Spectra
Source: PLoS Genet. 2017 Jan 19;13(1):e1006570. doi: 10.1371/journal.pgen.1006570 (PMC5289635; doi:10.1371/journal.pgen.1006570)
Supplement: S5 Table — (DOCX) [file pgen.1006570.s007.docx]

**S5 Table. Expression values of *E. coli* REL4536 genes involved in DNA repair, replication and transposition.**

| Gene | Description | log2(Fold Change)^†^ | Fold Change^††^ | BH adj. *p-*values* |
| --- | --- | --- | --- | --- |
| **Base excision repair pathway** | | | | |
| *alkA* | 3-methyl-adenine DNA glycosylase II | -0.88 | 0.54 | 1.29 × 10^-5^ |
| *mug/ygjF* | Stationary phase uracil DNA glycosylase | -0.69 | 0.62 | 1.76 × 10^-3^ |
| *mutM* | Formamidopyrimidine DNA glycosylase | 1.16 | 2.23 | 3.34 × 10^-5^ |
| *mutT* | Nucleotide hydrolysis glycosylase | 0.13 | 1.10 | 0.78 |
| *mutY* | Adenine DNA glycosylase | -0.90 | 0.53 | 5.58 × 10^-6^ |
| *nei* | Endonuclease VIII | 0.20 | 1.15 | 0.52 |
| *nfi* | Endonuclease V | 2.04 | 4.12 | 1.92 × 10^-17^ |
| *nfo* | Endonuclease IV | 1.06 | 2.08 | 5.26 × 10^-6^ |
| *Nth* | Endonuclease III | 1.39 | 2.63 | 8.29 × 10^-6^ |
| *tag* | 3-methyl-adenine DNA glycosylase I | 0.06 | 1.04 | 0.84 |
| *ung* | Uracil DNA glycosylase | 1.14 | 2.21 | 1.99 × 10^-9^ |
| *xthA* | Exonuclease III | 0.64 | 1.56 | 8.46 × 10^-3^ |
| **Mismatch repair pathway** | | | | |
| *dam* | DNA adenine methyltransferase | 1.27 | 2.41 | 3.45 × 10^-10^ |
| *exoX* | Exonuclease X | 1.31 | 2.48 | 1.27 × 10^-12^ |
| *mutH* | Endonuclease MutH | 1.02 | 2.03 | 8.98 × 10^-4^ |
| *mutL* | Molecular matchmaker MutL | 0.01 | 1.01 | 0.97 |
| *mutS* | Repair initiator MutS | 2.58 | 5.97 | 4.44 × 10^-29^ |
| *recJ* | Exonuclease RecJ | 0.41 | 1.33 | 0.05 |
| *sbcB/ExoI* | Exonuclease I | 1.05 | 2.07 | 5.94 × 10^-9^ |
| *ssb* | Single-stranded DNA binding protein | -1.08 | 0.47 | 2.99 × 10^-9^ |
| *xseA* | Exonuclease VII | -0.77 | 0.59 | 1.66 × 10^-3^ |
| *xseB/*  *ExoVII* | Exonuclease VII | 2.14 | 4.42 | 3.86 × 10^-25^ |
| *ybcN* | G:T mismatch repair protein | -0.86 | 0.55 | 0.16 |
| **Nucleotide excision repair pathway** | | | | |
| *cho/ydjQ* | DNA repair endonuclease also SOS inducible | 1.53 | 2.90 | 2.65 × 10^-16^ |
| *mfd* | DNA repair endonuclease | 1.54 | 2.90 | 5.73 × 10^-17^ |
| *uvrA* | UvrA exinuclease | 0.59 | 1.51 | 2.67 × 10^-3^ |
| *uvrB* | UvrB exinuclease | 0.31 | 1.24 | 0.09 |
| *uvrC* | UvrC exinuclease | -0.35 | 0.79 | 0.07 |
| *uvrD* | Helicase II, also involved in mismatch repair | 1.07 | 2.09 | 1.51 × 10^-8^ |
| **Recombinational repair** | | | | |
| *helD* | DNA helicase IV in RecF pathway | -1.81 | 0.29 | 2.96 × 10^-18^ |
| *intR* | Integrase | -1.52 | 0.35 | 3.88 × 10^-13^ |
| *pepA* | Aminopeptidase A/I | -1.04 | 0.49 | 8.82 × 10^-10^ |
| *recA* | DNA recombination protein | -0.04 | 0.98 | 0.88 |
| *recB* | Component of the RecBCD complex | 1.01 | 2.01 | 8.29 × 10^-7^ |
| *recC* | Component of the RecBCD complex | 0.68 | 1.60 | 2.26 × 10^-4^ |
| *recD* | Component of the RecBCD complex | 0.52 | 1.43 | 0.03 |
| *recF* | DNA helicase | -0.24 | 0.85 | 0.37 |
| *recG* | DNA helicase for double stranded DNA repair | -0.48 | 0.72 | 0.05 |
| *recN* | Protein involved in DSB repair | 1.21 | 2.32 | 7.08 × 10^-9^ |
| *recO* | Component of RecFOR complex | 0.49 | 1.41 | 0.08 |
| *recQ* | DNA helicase involved in RecF recombination | 1.34 | 2.52 | 1.6 × 10^-7^ |
| *recR* | Component of RecFOR complex | -1.49 | 0.35 | 2.59 × 10^-11^ |
| *recT* | Recombinase in RecE recombination pathway | 1.54 | 2.91 | 1.1 × 10^-5^ |
| *rus* | Holliday junction endonuclease | 0.49 | 1.41 | 0.55 |
| *ruvA* | Component of RuvAB complex | 1.32 | 2.50 | 2.13 × 10^-10^ |
| *ruvB* | Component of RuvAB complex | 0.35 | 1.27 | 0.10 |
| *ruvC* | Holliday junction nuclease | 0.61 | 1.52 | 0.01 |
| *sbcC* | Double stranded DNA exonuclease | 1.35 | 2.55 | 1.26 × 10^-9^ |
| *sbcD* | Double stranded DNA exonuclease | 1.50 | 2.83 | 9.57 × 10^-10^ |
| *topB* | DNA topoisomerase III | 0.80 | 1.74 | 2.81 × 10^-4^ |
| *xerC* | Recombinase protein XerC | 1.89 | 3.70 | 3.28 × 10^-16^ |
| *xerD* | Recombinase protein XerD | -0.29 | 0.82 | 0.15 |
| *yigN* | Putative recombination limiting protein | 2.48 | 5.59 | 7.79 × 10^-41^ |
| *yqgF* | Predicted transcription antitermination factor | 1.38 | 2.60 | 2.73 × 10^-6^ |
| **Transposition** | | | | |
| *insA* | IS1 protein | 0.32 | 1.25 | 0.09 |
| *insB* | IS1 protein | 1.30 | 2.46 | 9.6 × 10^-14^ |
| *insC* | IS2 protein | 0.28 | 1.22 | 0.69 |
| *insD* | IS2 protein | 0.43 | 1.35 | 0.19 |
| *insE* | IS3 protein | -0.69 | 0.62 | 8.4 × 10^-4^ |
| *insF* | IS3 protein | 0.35 | 1.28 | 0.07 |
| *insG* | IS4 protein | -0.79 | 0.58 | 1.5 × 10^-4^ |
| *insI* | IS30 protein | 0.60 | 1.52 | 0.03 |
| *insJ* | IS150 protein | 0.95 | 1.94 | 1.1 × 10^-4^ |
| *insK* | IS150 protein | 0.72 | 1.65 | 7 × 10^-4^ |
| *insL* | IS186 protein | -0.08 | 0.95 | 0.72 |
| *insN* | IS911 protein | 0.96 | 1.95 | 1.35 × 10^-3^ |
| *ECB_04146* | IS911 protein | 0.75 | 1.68 | 7.72 × 10^-3^ |
| *yis1* | IS600 protein | 0.28 | 1.22 | 0.69 |
| *yis2* | IS600 protein | 0.14 | 1.10 | 0.59 |
| **DNA replication** | | | | |
| *dinB* | DNA polymerase IV for translesion synthsis | -0.04 | 0.97 | 0.86 |
| *dinG* | Helicase, SOS inducible | 0.64 | 1.55 | 2.57 × 10^-3^ |
| *dnaA* | Replication initiation protein | 0.31 | 1.24 | 0.13 |
| *dnaB* | DNA helicase | 2.63 | 6.17 | 3.51 × 10^-40^ |
| *dnaC* | DNA replication initiation protein | -0.74 | 0.60 | 3.85 × 10^-4^ |
| *dnaE* | DNA polymerase III | -0.34 | 0.79 | 0.09 |
| *dnaG* | DNA primase | -1.29 | 0.41 | 5.02 × 10^-14^ |
| *dnaJ* | DNA chaperone protein | 1.64 | 3.12 | 1.94 × 10^-18^ |
| *dnaK* | DNA chaperone protein | 2.41 | 5.30 | 2.77 × 10^-50^ |
| *dnaN* | DNA polymerase III beta subunit | 0.01 | 1.01 | 0.97 |
| *dnaQ* | DNA polymerase III epsilon subunit | -0.62 | 0.65 | 7.44 × 10^-4^ |
| *dnaT* | Replication protein, SOS inducible | -0.94 | 0.52 | 7.32 × 10^-6^ |
| *dnaX* | DNA polymerase III gamma&tau subunit | 1.72 | 3.29 | 2.95 × 10^-20^ |
| *hda* | Replication initiation factor | 1.24 | 2.36 | 2.77 × 10-5 |
| *holA* | DNA polymerase III subunit | 0.58 | 1.49 | 0.02 |
| *holB* | DNA polymerase III delta subunit | 1.02 | 2.03 | 1.77 × 10^-6^ |
| *holC* | DNA polymerase III subunit | -0.11 | 0.93 | 0.63 |
| *holD* | DNA polymerase III subunit | 2.32 | 4.99 | 7.14 × 10^-21^ |
| *holE* | DNA polymerase III theta subunit | 1.83 | 3.55 | 2.33 × 10^-14^ |
| *ligA* | DNA ligase | -1.29 | 0.41 | 7.44 × 10^-13^ |
| *ligB* | DNA ligase | 1.14 | 2.21 | 4.53 × 10^-7^ |
| *mukB* | Cell division protein involved in partitioning | 0.13 | 1.09 | 0.54 |
| *mukE* | Cell division protein involved in partitioning | 2.01 | 4.02 | 1.11 × 10^-21^ |
| *mukF* | Cell division protein involved in partitioning | 1.31 | 2.48 | 1.69 × 10^-7^ |
| *nrdA* | Ribonucleoside diphosphate reductase 1 | 0.84 | 1.79 | 1.89 × 10^-4^ |
| *nrdB* | Ribonucleoside diphosphate reductase 1 | 0.59 | 1.50 | 9.65 × 10^-3^ |
| *nrdD* | Ribonucleoside-triphosphate | 1.20 | 2.30 | 2.87 × 10^-7^ |
| *nrdE* | Ribonucleoside-diphosphate reductase 2 | 1.29 | 2.45 | 2.84 × 10^-10^ |
| *nrdF* | Ribonucleoside-diphosphate reductase 2 | 1.03 | 2.04 | 2.54 × 10^-5^ |
| *pioO* | Calcium-binding protein | 0.48 | 1.40 | 0.07 |
| *polA* | DNA polymerase I | -0.55 | 0.68 | 4.3 × 10^-3^ |
| *polB* | DNA polymerase II for translesion synthsis | 1.28 | 2.43 | 2.9 × 10^-9^ |
| *priA* | Primosomal replication factor | 0.50 | 1.42 | 0.03 |
| *priB* | Primosomal replication factor | -0.73 | 0.60 | 0.05 |
| *priC* | Primosomal replication factor | -0.14 | 0.91 | 0.60 |
| *rep* | Helicase that prevents DSBs | 2.10 | 4.29 | 1.17 × 10^-15^ |
| *seqA* | Replication regulator | 2.49 | 5.61 | 1.51 × 10^-38^ |
| *tdk* | Thymidine kinase/deoxyuridine kinase | -1.28 | 0.41 | 2.56 × 10^-6^ |
| *tus* | Replication terminator | -1.88 | 0.27 | 1.52 × 10^-25^ |
| *umuC* | DNA polymerase V subunit | 0.38 | 1.30 | 0.17 |
| *umuD* | DNA polymerase V subunit | 0.39 | 1.31 | 0.11 |
| *ycaJ* | Protein involved in recombination | 0.44 | 1.35 | 0.03 |
| *yraO* | DnaA initiator-associating factor | 0.31 | 1.24 | 0.19 |
| **SOS response** | | | | |
| *dinD* | DNA damage inducible protein | 1.79 | 3.46 | 1.7 × 10^-14^ |
| *dinF* | Member of the MATE family of multidrug efflux transporters | 0.39 | 1.31 | 0.23 |
| *dinI* | DNA damage-inducible protein I | -0.32 | 0.80 | 0.37 |
| *ftsK* | Cell division protein | -0.15 | 0.90 | 0.40 |
| *lexA* | Transcription repressor LexA | -1.06 | 0.48 | 3.89 × 10^-5^ |
| *recX* | Inhibitor of RecA | -0.09 | 0.94 | 0.84 |
| *rimK* | ribosomal protein S6 modification protein | -1.25 | 0.42 | 5.25 × 10^-7^ |
| *sulA* | Cell division inhibitor SulA | -1.96 | 0.26 | 2.26 × 10^-27^ |
| *yafP* | Predicted acyltransferase with acyl-CoA N-acyltransferase domain | 0.64 | 1.56 | 8.46 × 10^-3^ |
| *ydjM* | Predicted inner membrane protein, | -0.61 | 0.65 | 0.01 |
| *yebG* | DNA damage-inducible protein | 0.30 | 1.23 | 0.19 |
| *yjiW* | Toxin-like protein of the SOS response | 1.63 | 3.09 | 2.33 × 10^-13^ |
| **Stringent response** | | | | |
| *ahpC* | Alkyl hydroperoxide reductase | 0.82 | 1.77 | 8.92 × 10^-7^ |
| *appA* | Acid phosphatase | 0.77 | 1.70 | 5.47 × 10^-5^ |
| *appY* | DNA-binding transcriptional activator | -0.05 | 0.97 | 0.94 |
| *glnG* | NtrC transcriptional dual regulator | -1.08 | 0.47 | 7.84 × 10^-8^ |
| *leuB* | 3-isopropylmalate dehydrogenase | 0.34 | 1.27 | 0.61 |
| *mazG* | Nucleoside triphosphate pyrophosphohydrolase | 1.15 | 2.22 | 3.24 × 10^-9^ |
| *phoQ* | Sensory histidine kinase | -0.39 | 0.76 | 0.07 |
| *phoR* | Sensory histidine kinase | -0.34 | 0.79 | 0.18 |
| *phoU* | Negative regulator of the *Pho* regulon | 0.25 | 1.19 | 0.39 |
| *rplK* | 50S ribosomal subunit protein L11 | 1.14 | 2.20 | 1.06 × 10^-8^ |
| *rpmE2* | 50S ribosomal protein L31 type B | -1.80 | 0.29 | 8.26 × 10^-6^ |
| *ssuE* | NAD(P)H-dependent FMN reductase | 0.72 | 1.65 | 0.14 |
| *tas* | Putative NAD(P)-linked reductase that acts in starvation-associated mutation | 1.90 | 3.73 | 8.44 × 10^-17^ |
| *tauA* | Taurine ABC transporter - periplasmic binding protein | 0.30 | 1.23 | 0.22 |
| *tauB* | Taurine ABC transporter - ATP binding subunit | 0.56 | 1.47 | 0.04 |
| *tauC* | Taurine ABC transporter - membrane subunit | -0.51 | 0.70 | 0.10 |
| *yaiB* | Sigma factor inhibitor | -1.54 | 0.34 | 7.1 × 10^-9^ |
| *ycgW* | Iron-sulfur cluster scaffold protein | 0.99 | 1.99 | 0.13 |
| *yhgI* |  | 0.80 | 1.75 | 2.5 × 10^-4^ |
| *yibD* | UDP-glucuronate:LPS(HepIII) glycosyltransferase | -0.57 | 0.67 | 0.04 |
| *yjbA* | Predicted phosphate starvation-inducible protein | 1.25 | 2.38 | 1 × 10^-5^ |
| *yjiY* | Inner membrane protein | 1.04 | 2.06 | 4.48 × 10^-7^ |
| *yodA* | Cadmium-induced cadmium binding protein | -1.28 | 0.41 | 1.03 × 10^-5^ |

^†^DESeq2 outputs log2(Fold Change) values calculated from normalized sequence read count data.

^††^As a descriptive detail, fold change was calculated as the ratio of a gene’s mean expression in anaerobic conditions to its mean expression in aerobic conditions. A fold change greater than one indicates greater expression in anaerobic conditions, while a fold change between zero and one indicates greater expression under aerobic conditions.

^*^Benjamini-Hochberg (BH) adjusted *p*-values as implemented in the DESeq2 package to control the False Discovery Rate (FDR). An FDR threshold < 0.05 was used to identify significant expression.
