# Supplementary material for: Anaerobically Grown Escherichia coli Has an Enhanced Mutation Rate and Distinct Mutational Spectra
Source: PLoS Genet. 2017 Jan 19;13(1):e1006570. doi: 10.1371/journal.pgen.1006570 (PMC5289635; doi:10.1371/journal.pgen.1006570)
Supplement: S6 Table — (DOCX) [file pgen.1006570.s008.docx]

**S6 Table. Relative abundance of IS gene transcripts in aerobic and anaerobically grown cells.**

| Gene | Product | Gene length (bp) | Mean relative transcript abundance in aerobically grown cells^†^ | Mean relative transcript abundance in anaerobically grown cells^†^ |
| --- | --- | --- | --- | --- |
| *insA* | IS1 protein | 276 | 2.34 × 10^-6^ | 5.42 × 10^-6^ |
| *insB* | IS1 protein | 504 | 4.41 × 10^-7^ | 2.01 × 10^-6^ |
| *insC* | IS2 protein | 261 | 7.84 × 10^-9^ | 1.88 × 10^-8^ |
| *insD* | IS2 protein | 906 | 1.24 × 10^-8^ | 3.13 × 10^-8^ |
| *insE* | IS3 protein | 309 | 3.02 × 10^-7^ | 3.43 × 10^-7^ |
| *insF* | IS3 protein | 867 | 2.08 × 10^-7^ | 4.93 × 10^-7^ |
| *insG* | IS4 protein | 1329 | 1.02 × 10^-7^ | 1.10 × 10^-7^ |
| *insI* | IS30 protein | 1125 | 1.49 × 10^-8^ | 4.20 × 10^-8^ |
| *insJ* | IS150 protein | 522 | 6.33 × 10^-8^ | 2.27 × 10^-7^ |
| *insK* | IS150 protein | 852 | 3.50 × 10^-7^ | 1.06 × 10^-6^ |
| *insL* | IS186 protein | 1113 | 1.79 × 10^-7^ | 3.15 × 10^-7^ |
| *insN* | IS911 protein | 273 | 4.12 × 10^-8^ | 1.51 × 10^-7^ |
| *ECB_04146* | IS911 protein | 840 | 1.90 × 10^-8^ | 5.90 × 10^-8^ |
| *yis1* | IS600 protein | 303 | 6.92 × 10^-9^ | 1.62 × 10^-8^ |
| *yis2* | IS600 protein | 819 | 4.19 × 10^-8^ | 8.55 × 10^-8^ |

^†^Values normalized for gene length.
