# Supplementary material for: Anaerobically Grown Escherichia coli Has an Enhanced Mutation Rate and Distinct Mutational Spectra
Source: PLoS Genet. 2017 Jan 19;13(1):e1006570. doi: 10.1371/journal.pgen.1006570 (PMC5289635; doi:10.1371/journal.pgen.1006570)
Supplement: S7 Table — (DOCX) [file pgen.1006570.s009.docx]

**S7 Table. IS*150* genome location.**

| IS*150* copy^†^ | Genome location | Orientation^††^ | Disrupted ORF | Left ORF (orientation^††^) | Right ORF (orientation^††^) | Comment |
| --- | --- | --- | --- | --- | --- | --- |
| 6 | 998,183 - 999,625 | - | *pykF* (-) | *lpp* (-) | *ydhZ (+)* | Disruption of pyruvate kinase I gene. |
| 7 | 1,764,886 - 1,766,328 | - | *pflB* (+) | *focA* (+) | *pflA* (+) | Disruption of pyruvate formate lyase gene. |
| 2 | 2,070,037 - 2,071,479 | - | intergenic | *gltK* (+) | *rihA* (+) | A previously unrecognised gene, predicted to encode an arginine ABC transporter ATP-binding protein, has been disrupted. This is predicted to be within the *glt* operon, located downstream of *gltK.* |
| 3 | 2,747,257 - 2,748,699 | - | intergenic | *insL-5* (+) | *cysH* (-) | The *cys* operon is involved in sulphate reduction. |
| 4 | 3,623,897 - 3,625,339 | + | intergenic | *hokA* (-) | *glyS* (-) | IS orientation is in reverse to that of adjacent genes, *glyS* encodes subunit of glycine tRNA ligase. |
| 5 | 3,866,362 - 3,867,804 | + | intergenic | *trkD* (+) | *yieP* (-) | The *trkD* gene is involved in potassium ion uptake. |

^†^IS*150* copy number. Copy 1 (not shown) is a partial IS*150* element that has been disrupted by IS*1* in *insK-1*, and is thus not included in this table.

^††^Orientation, shown as + or -, of IS*150* (or gene) relative to the top strand of the reference genome sequence.
