# Supplementary material for: Anaerobically Grown Escherichia coli Has an Enhanced Mutation Rate and Distinct Mutational Spectra
Source: PLoS Genet. 2017 Jan 19;13(1):e1006570. doi: 10.1371/journal.pgen.1006570 (PMC5289635; doi:10.1371/journal.pgen.1006570)
Supplement: S8 Table — (DOCX) [file pgen.1006570.s010.docx]

**S8 Table. Clockwise and counter-clockwise replichores of *E. coli* REL4536.**

|  |  | Clockwise replichore  (template leading strand) | Counter-clockwise replichore  (template lagging strand) |
| --- | --- | --- | --- |
| Size (bp) |  | 2,055,789 | 2,539,896 |
| Nucleotide composition |  |  |  |
|  | No. of A (bp) | 504,576 (24.5%) | 627,644 (24.7%) |
|  | No. of C (bp) | 506,731 (24.6%) | 666,344 (26.2%) |
|  | No. of G (bp) | 536,404 (26.1%) | 625,390 (24.6%) |
|  | No. of T (bp) | 508,078 (24.7%) | 620,518 (24.4%) |
| No. of CDS |  | 1,853 | 2,323 |
|  | No. of co-directional CDS | 858 | 1,291 |
|  | No. of head on CDS | 995 | 1,032 |
