# Supplementary material for: Anaerobically Grown Escherichia coli Has an Enhanced Mutation Rate and Distinct Mutational Spectra
Source: PLoS Genet. 2017 Jan 19;13(1):e1006570. doi: 10.1371/journal.pgen.1006570 (PMC5289635; doi:10.1371/journal.pgen.1006570)
Supplement: S9 Table — (DOCX) [file pgen.1006570.s011.docx]

**S9 Table. Expression of genes involved in arginine biosynthesis.**

| Gene | Product | log2(Fold Change) ^†^ | Fold Change^††^ | BH adj. *p-*values* |
| --- | --- | --- | --- | --- |
| *yhaO* | Putative transporter | 5.77 | 54.53 | 1.64 × 10^-194^ |
| *artJ* | Arginine transporter subunit | 5.56 | 47.21 | 2.85 × 10^-149^ |
| *argA* | *N*-acetylglutamate synthase | 3.82 | 14.13 | 3.23 × 10^-77^ |
| *ybiA* | Swarming motility protein | 3.69 | 12.86 | 1.44 × 10^-60^ |
| *argH* | Argininosuccinate lyase | 3.65 | 12.53 | 7.63 × 10^-64^ |
| *argC* | *N*-acetyl-gamma-glutamyl-phosphate | 3.60 | 12.10 | 8.14 × 10^-86^ |
| *purK* | *N*5-carboxyaminoimidazole ribonucleotide synthase | 3.59 | 12.01 | 3.43× 10^-24^ |

^†^DESeq2 outputs log2(Fold Change) values calculated from normalized sequence read count data.

^††^As a descriptive detail, fold change was calculated as the ratio of a gene’s mean expression in anaerobic conditions to its mean expression in aerobic conditions. A fold change greater than one indicates greater expression in anaerobic conditions, while a fold change between zero and one indicates greater expression under aerobic conditions.

^*^Benjamini-Hochberg (BH) adjusted *p*-values as implemented in the DESeq2 package to control the False Discovery Rate (FDR). An FDR threshold < 0.05 was used to identify significant expression.
