# Supplementary material for: Anaerobically Grown Escherichia coli Has an Enhanced Mutation Rate and Distinct Mutational Spectra
Source: PLoS Genet. 2017 Jan 19;13(1):e1006570. doi: 10.1371/journal.pgen.1006570 (PMC5289635; doi:10.1371/journal.pgen.1006570)
Supplement: S10 Table — (DOCX) [file pgen.1006570.s012.docx]

**S10 Table. Primers used to verify SV break points**.

| Primer name | Sequence (5' 🡪 3') | PCR annealing temperature |
| --- | --- | --- |
| IS150 insertion F | CGT TGT CTC TCG TCC AGG TT | 51ºC |
| IS150 insertion R | AGG CGG CAA ATT TGT CTG TA | 51ºC |
| 6 kb deletion within F | TCC GGA AGA ACT GGC TCT AA | 52ºC |
| 6 kb deletion within R | GGA ATC AGC ACC GAC AAT TT | 52ºC |
| ybdk F | CCT GAA TTA ATC CCG CCA TA | 52ºC |
| entD R | AGC CGT GGT ATC TCG TCA AC | 57ºC |
| nupC F | GGA TTA TCG CAG GTG CAG TT | 56ºC |
| yfeaA R | GCA TTT ATT CTT GCG GTG CT | 54ºC |
| clpX F | ATC TGG AAT TCC GTG ACG AG | 55ºC |
| lon R | ATC GCG TTT ATT TTC GAA CG | 52ºC |
| ynhG F | GGC GAA TAC CTC ATT CAT GG | 53ºC |
| ydhY R | AGG TGT CCG CGG TAT AGT TG | 57ºC |
| gltK F | TGG AGT TCC ATT GTC CCT TC | 55ºC |
| rihA R | GGA TCC CTG ACT GGA AGA CA | 56ºC |
| focA F | GCT TTC CGG CGA GTA TAT GA | 55ºC |
| pflA R | TGC CCA TAT CAC GGG TAA AT | 54ºC |
| ybdB F | CAC CAG CGA TAA CAC AAT GG | 54ºC |
| ybdH R | GGT GTG GAT CTA CGG CAA AC | 56ºC |
| iap F | AGT TTT CGA CAA AGC CGG TA | 54ºC |
| cysH R | GCA AAA ACA TGG CCT GAA AT | 52ºC |
| trkD F | GCT GGT GAT TAT GGG GCT AA | 54ºC |
| hdfR R | GGC CAG ATT TTC AAC AGC AT | 54ºC |
| yeaS F | ACG CTG GAA CTG GTG AGT TT | 57ºC |
| yeaR R | ACT TCA GGA GCC ACG AAG AA | 56ºC |
| gmhB F | GTG GGA ACA AAA GTG CTG GT | 56ºC |
| dkgB R | TAT GGA TGC CGT GCT GTT TA | 54ºC |
| kgtP F | CTC ACT ATC AGC AGG GCA CA | 57ºC |
| clpB R | GAA GGC ATC GCT TTC TGG TA | 55ºC |
| yibF F | AGT CGC CAG ATT GAC CGT AT | 56ºC |
| yibA R | CGT CTT GCC CAC CTC TTA AC | 56ºC |
| nikR F | CGT GTT GAA AGG TGA CAT GG | 54ºC |
| yhhJ R | GAA AGT ATG CCG CAG ATG GT | 55ºC |
| ybfO F | CGG ATT CAG CGG ATA CTG AT | 54ºC |
| kdpA R | CGG CAG AAA GAA AGT TTT GC | 53ºC |
| ybbP F | GCT TAA CCG CGA ACT CAA TC | 54ºC |
| ylbH R | GTG ATG CGG GTT CTC TTC AT | 55ºC |
| ECB_01416 F | AGC AAC TCT GGA ATG GCT GT | 57ºC |
| ECB_01413 R | ACC GCA CCA CTG ATG TGA TA | 56ºC |
