# Supplementary figures and images for: Anaerobically Grown Escherichia coli Has an Enhanced Mutation Rate and Distinct Mutational Spectra
Source: PLoS Genet. 2017 Jan 19;13(1):e1006570. doi: 10.1371/journal.pgen.1006570 (PMC5289635; doi:10.1371/journal.pgen.1006570)

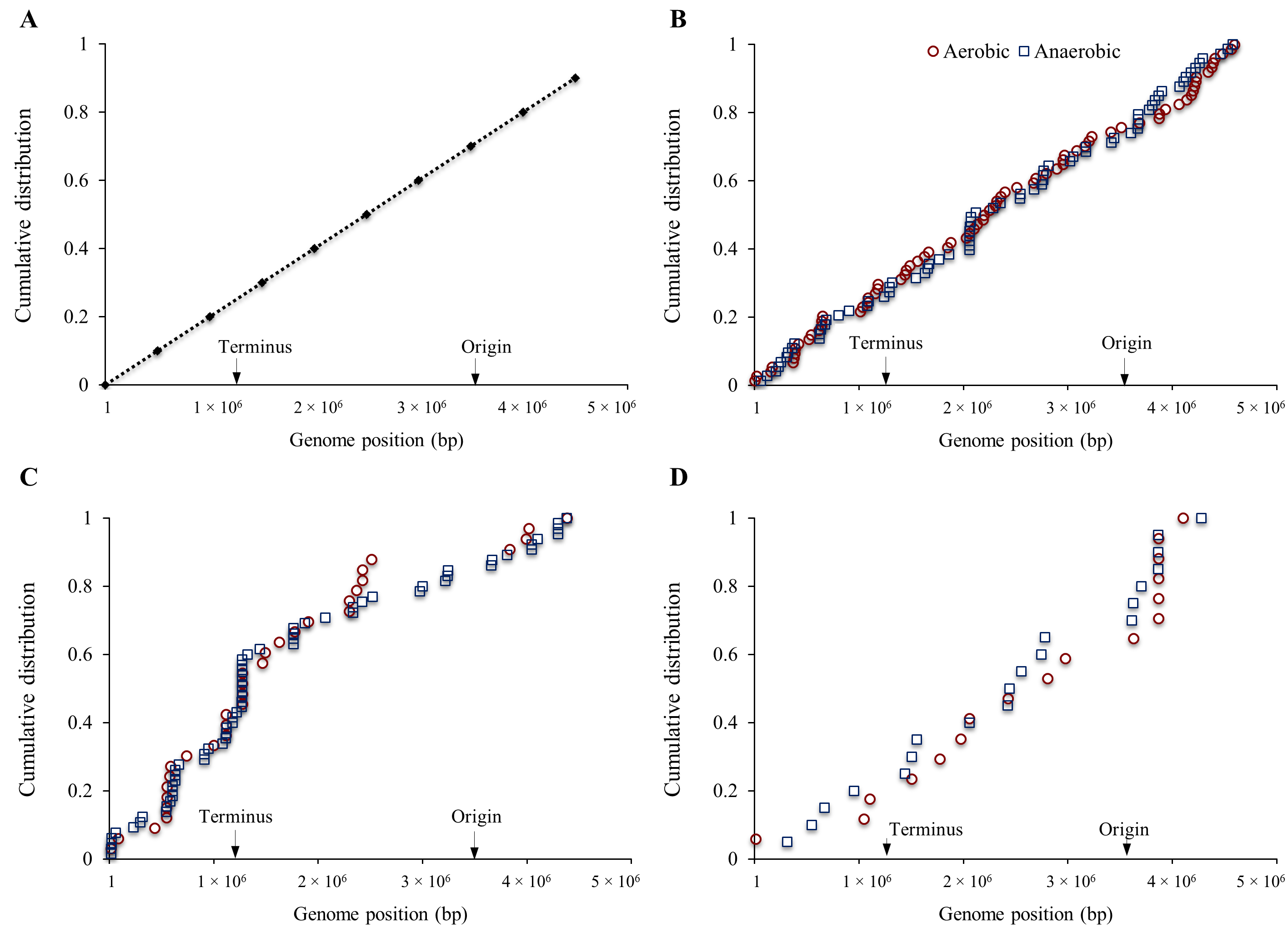

Supplement: S1 Fig — Shown are the relationships between cumulative mutations and chromosomal position for A) expected random distribution of mutations around the genome, B) BPSs, C) SVs, and D) indels. (TIF) [file pgen.1006570.s013.tif]

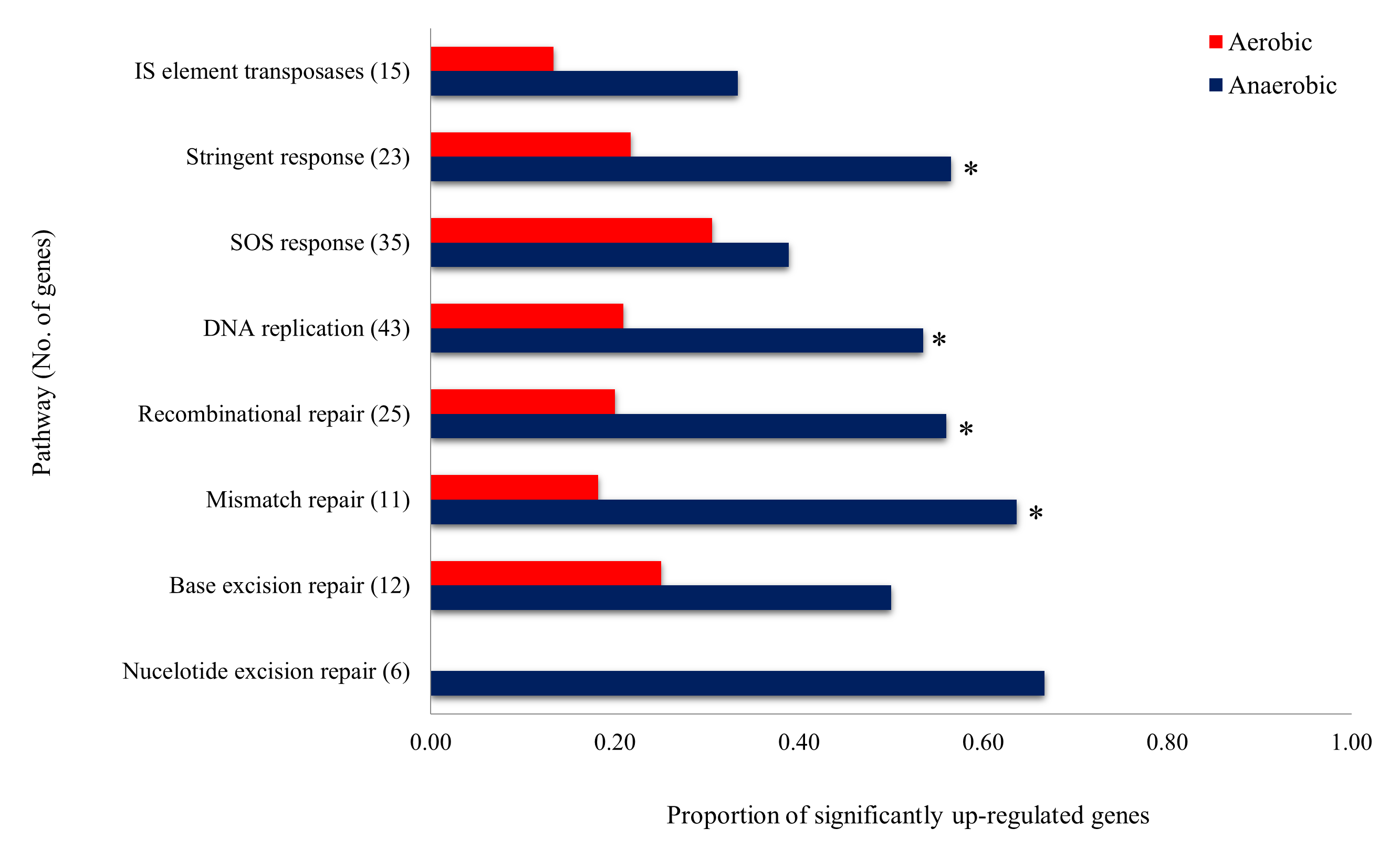

Supplement: S2 Fig — To identify significant expression, Benjamini-Hochberg adjusted p < 0.05 were used. Asterisk denotes a significant enrichment of the gene list under anaerobic conditions by Fisher’s exact test (*, p < 0.05). (TIF) [file pgen.1006570.s014.tif]

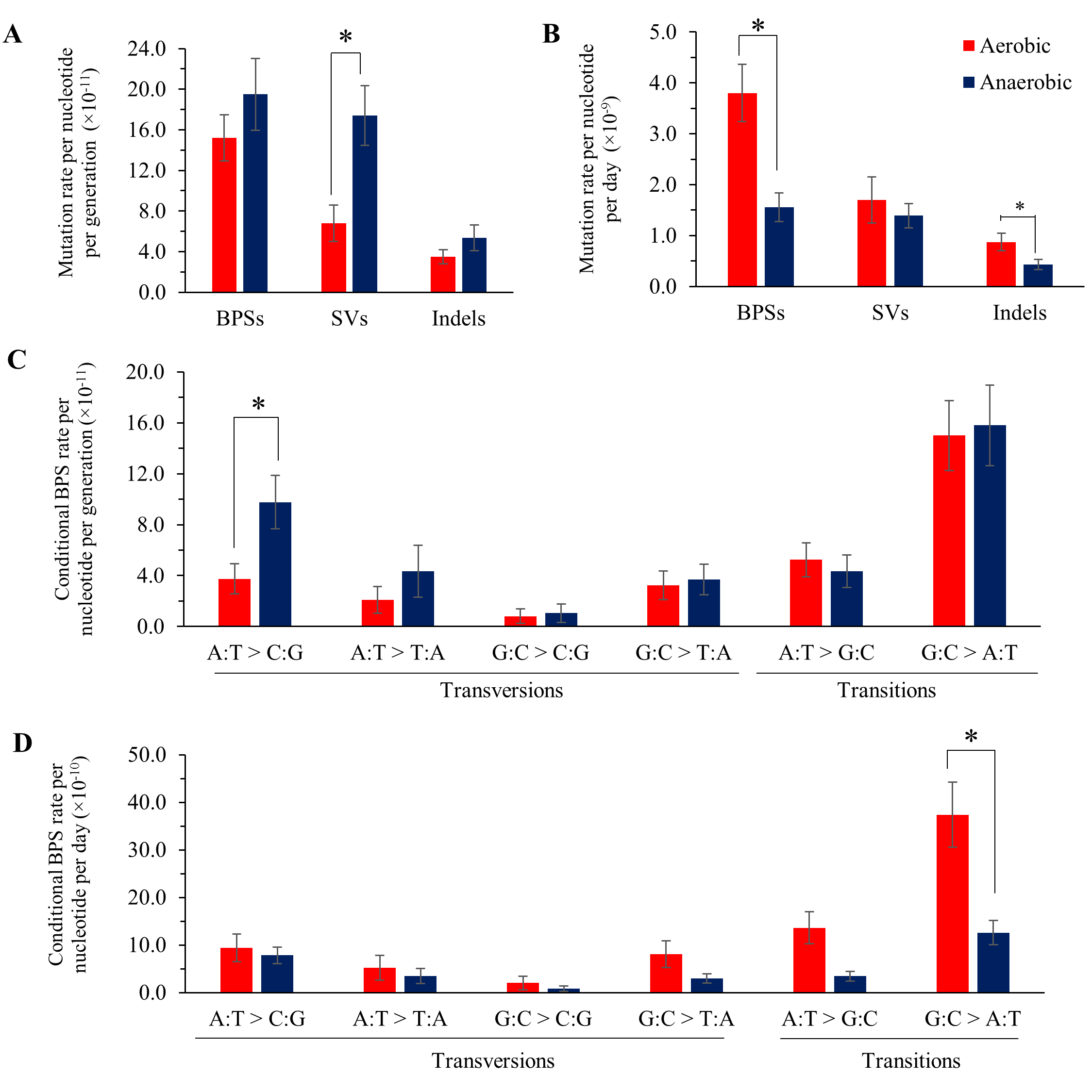

Supplement: S3 Fig — Shown are: Mean mutation rates of mutational classes expressed A) per nucleotide per generation, B) per nucleotide per day of growth, C) Conditional BPS rates expressed per nucleotide per generation and D) Conditional BPS rates expressed per nucleotide per day of growth. Error bars represent standard errors of the mean. * denotes p < 0.05 by Mann-Whitney U-test. (TIF) [file pgen.1006570.s015.tif]

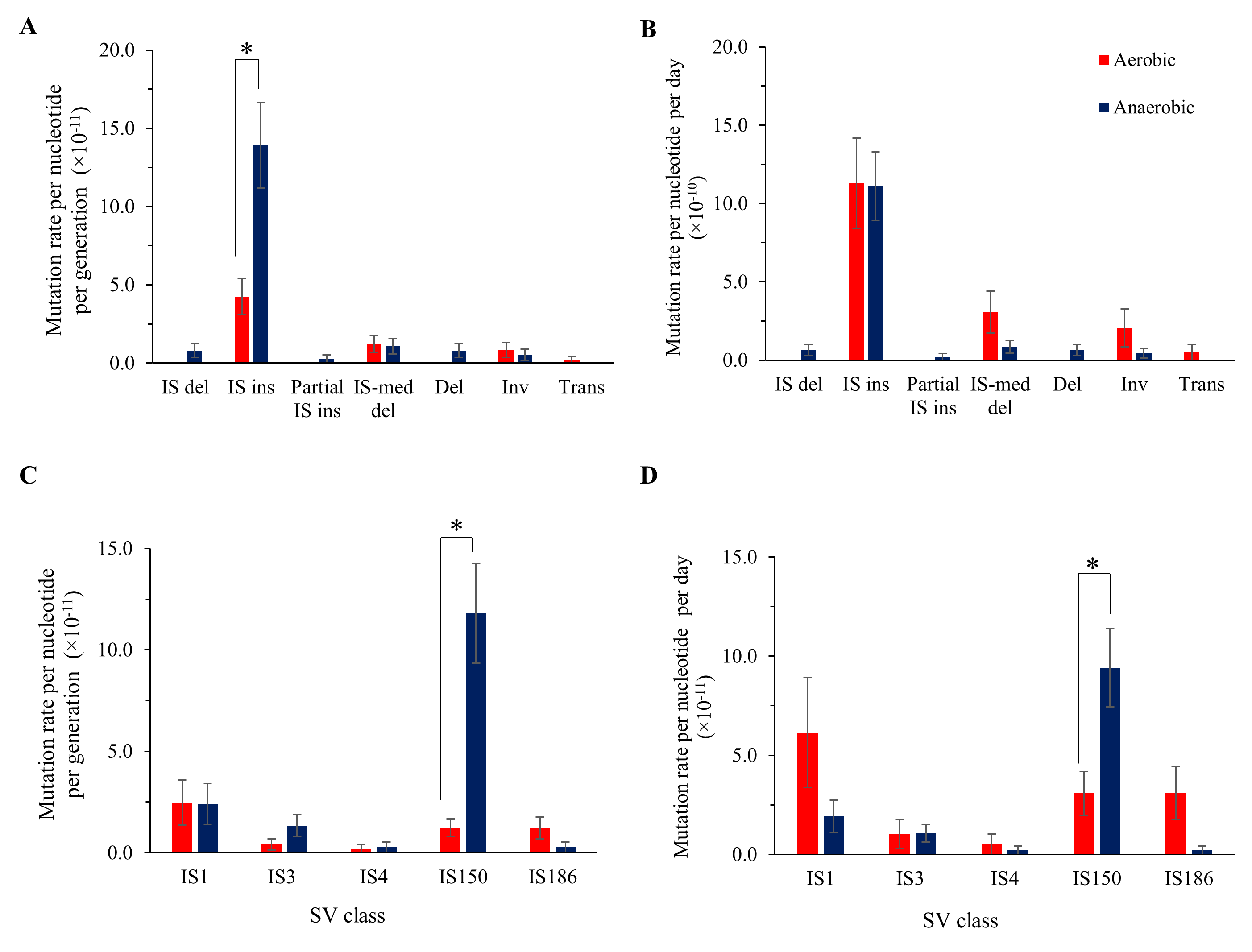

Supplement: S4 Fig — Shown are: A) and C) Mutation rates of different SV classes expressed per nucleotide per generation, and B) and D) Mutation rates of different SV classes expressed per nucleotide per day of growth. Error bars represent standard errors of the mean. * denotes p < 0.05 by Mann-Whitney U-test. (TIF) [file pgen.1006570.s016.tif]
